# The geomicrobiology of limestone, sulfuric acid speleogenetic, and volcanic caves: basic concepts and future perspectives

**DOI:** 10.3389/fmicb.2024.1370520

**Published:** 2024-03-20

**Authors:** Paolo Turrini, Alif Chebbi, Filippo Pasquale Riggio, Paolo Visca

**Affiliations:** ^1^Department of Science, Roma Tre University, Rome, Italy; ^2^National Biodiversity Future Center, Palermo, Italy

**Keywords:** geomicrobiology, lava tube cave, limestone cave, microbial diversity, microbiome, sulfuric acid speleogenetic cave, volcanic cave, metataxonomic profiling

## Abstract

Caves are ubiquitous subterranean voids, accounting for a still largely unexplored surface of the Earth underground. Due to the absence of sunlight and physical segregation, caves are naturally colonized by microorganisms that have developed distinctive capabilities to thrive under extreme conditions of darkness and oligotrophy. Here, the microbiomes colonizing three frequently studied cave types, i.e., limestone, sulfuric acid speleogenetic (SAS), and lava tubes among volcanic caves, have comparatively been reviewed. Geological configurations, nutrient availability, and energy flows in caves are key ecological drivers shaping cave microbiomes through photic, twilight, transient, and deep cave zones. Chemoheterotrophic microbial communities, whose sustenance depends on nutrients supplied from outside, are prevalent in limestone and volcanic caves, while elevated inorganic chemical energy is available in SAS caves, enabling primary production through chemolithoautotrophy. The 16S rRNA-based metataxonomic profiles of cave microbiomes were retrieved from previous studies employing the Illumina platform for sequencing the prokaryotic V3-V4 hypervariable region to compare the microbial community structures from different cave systems and environmental samples. Limestone caves and lava tubes are colonized by largely overlapping bacterial phyla, with the prevalence of *Pseudomonadota* and *Actinomycetota*, whereas the co-dominance of *Pseudomonadota* and *Campylobacterota* members characterizes SAS caves. Most of the metataxonomic profiling data have so far been collected from the twilight and transient zones, while deep cave zones remain elusive, deserving further exploration. Integrative approaches for future geomicrobiology studies are suggested to gain comprehensive insights into the different cave types and zones. This review also poses novel research questions for unveiling the metabolic and genomic capabilities of cave microorganisms, paving the way for their potential biotechnological applications.

## 1 Introduction

Caves are defined as underground voids accessible to humans (Morgan, 1991). They are present in various lands worldwide (Ford and Williams, 2007) and represent a huge reservoir of still unexplored biodiversity. Caves also provide a huge surface for interaction with colonizing microorganisms, serving as a model habitat to study the microbial communities living in the subsurface (Gillieson, 2021). Therefore, cave microbiology has emerged as a new field of geomicrobiology, continuously improving thanks to scientific and technological advances in environmental microbiome studies. The complexity of microbial life implicated in key processes of cave ecosystems, such as nutritional and biogeochemical cycles, has recently been highlighted (Zhu et al., 2022). Microorganisms constitute the majority of rock-associated biomass in caves, capable of colonizing any cave habitat, including extremely oligotrophic environments (Reboleira et al., 2022). The high microbial diversity in these habitats represents a unique source of new genetic, metabolic, and physiological information, with a potential impact on pharmaceutical and biotechnological applications (Zada et al., 2022).

From a geochemical point of view, caves significantly differ according to the type of rock substrate and the cave formation processes (White and Culver, 2019; De Waele and Gutierrez, 2022). The main mechanism is based on rock dissolution, and sedimentary carbonate rocks constitute the prevalent fraction of soluble rocks on the Earth. Karst is the name indicating a carbonate rock landscape and karst terrains represent 15.2 % of the global ice-free continental surface (Goldscheider et al., 2020).

Among carbonate rocks, those composed of calcite (CaCO_3_) are the prevalent substrate for limestone cave formation (Biagioli et al., 2023), as opposed to the less soluble dolomite [CaMg(CO_3_)_2_] rock. Limestone caves are often epigenic since generated by the acid rainwater actions that induce the dissolution of underlying calcite rocks (Culver and Pipan, 2009), while SAS caves are hypogenic since formed by hydrogen sulfide (H_2_S)-rich water rising from the depths of the Earth (Engel et al., 2004b; D’Angeli et al., 2019a). Other caves are formed by dissolution processes of different rock types. They include evaporite rocks (gypsum, halite, anhydrite), which are relatively young with very rapid morphological evolution (D’Angeli et al., 2017), and silicate caves, which require a very long formation time since they are embedded in rocks that are poorly soluble in water, such as sandstone and quartzite (Sauro et al., 2018; Ghezzi et al., 2022; Liu et al., 2022).

Pseudokarst caves comprise underground environments generated by mechanisms other than dissolution. Among these, volcanic caves (Kempe, 2019) including lava tubes are cavities originating from lava flows and movements (Northup and Lavoie, 2015), glacial caves formed from the action of meltwater flowing through or under the glaciers (Howarth, 2021), and man-made cavities, created by human excavation (Parise et al., 2013). Early studies on cave microbiology date back to the 1960s and have been conducted on water and sediments using basic microscopy and culture-based approaches (Caumartin, 1963). It was only in the 1990s that, following the advent of molecular technologies and the use of the 16S rRNA gene as a phylogenetic marker, it became possible to study microbial communities in more detail, by gaining comprehensive taxonomic information from environmental DNA (eDNA) extracted from cave samples (Engel et al., 2004a). Although the culturability of bacteria from caves remains very low (0.02–1%), some strains endowed with biotechnological potential have been isolated under laboratory conditions (Bender et al., 2020). Microorganisms ubiquitously colonize caves, though the microbial density is much less than that of the soil. For example, a single gram of soil harbors up to 10^10^ bacterial cells and an estimated species diversity of 4x10^3^ to 5x10^4^ species (Raynaud and Nunan, 2014). The microbial density in caves is estimated at <10^6^ microbial cells per gram of sample and varies depending on the distance from the entrance, the nutrient availability, and the rock geochemistry (Barton and Jurado, 2007; Barton, 2015).

Given the continuous improvement of next-generation sequencing (NGS) and analytical chemistry methods, various culture-independent approaches are being applied to cave microbiomes, e.g., 16S rRNA metataxonomic profiling (Biagioli et al., 2023), metagenomic shotgun sequencing (Wiseschart et al., 2019; Turrini et al., 2020), metatranscriptomics (Mondini et al., 2022) and proteomics (van Spanning et al., 2022). In parallel, high-magnification and fluorescence microscopies have contributed to unveiling biofilm-like structures on cave surfaces, identifying and quantifying specific taxa composing these communities, and studying the functional properties of individual microbial components (Jones et al., 2023). Cave microbiology has masterly been reviewed in the past, with an emphasis on geomicrobiology (Northup and Lavoie, 2001; Barton, 2006; Barton and Northup, 2007), anthropic impact (e.g., Bontemps et al., 2022), biogeochemical cycling (e.g., Zhu et al., 2022), biodiversity and functional roles of microbial communities (Engel, 2010; Kosznik-Kwaśnicka et al., 2022).

To the best of our knowledge, comparative studies of the microbial communities thriving in different types of caves (limestone, SAS, and volcanic caves) have not yet been conducted. Accordingly, here, we will *i*) describe the complexity of the cave ecosystem; *ii*) briefly overview basic concepts in cave microbiology; *iii*) describe the fundamental geological features of limestone, SAS, and volcanic caves, with a focus on the ecology and diversity of their microbial inhabitants; *iv*) compare the metataxonomic profiles of cave microbiomes retrieved from studies employing the Illumina (MiSeq) sequencing platform for sequencing the V3-V4 hypervariable regions of the prokaryotic 16S rRNA from limestone, SAS and lava tubes among volcanic caves; *v*) propose concepts and ideas for integrative cave microbiome studies, highlighting key steps of the investigation. We expect this review will also stimulate the exploitation of novel biotechnological potentials of cave microorganisms. The following three sections are intended to provide entry-level geomicrobiological information on the most common and best-studied cave ecosystems, to push forward and broaden the research interest into a still elusive component of the Earth’s microbiome.

## 2 The biological complexity of cave ecosystems

Caves are complex ecosystems comprising abiotic and biotic components (Culver and Pipan, 2009). All forms of life (i.e., viruses, bacteria, fungi, algae, protozoa, plants, and animals) have been described in subterranean ecosystems, including rock surfaces, groundwater pools, streams, bat guano, sediments, and others (Culver and Pipan, 2009).

**FIGURE 1:**
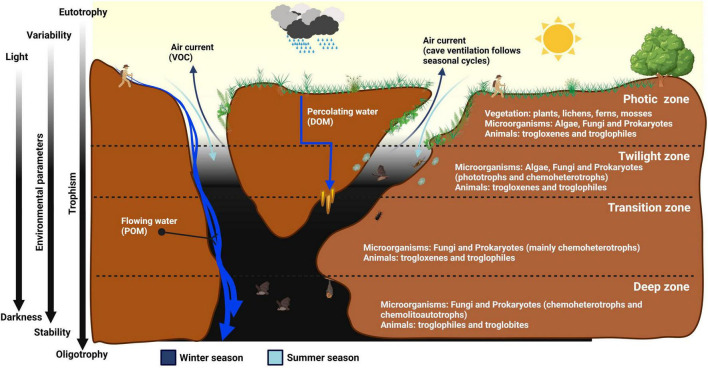
Schematic representation of the different cave zones, and their characteristics in relation to light intensity, temperature, humidity, airflow, and nutrient availability.

The cave fauna can be divided into three categories based on adaptation and cave-related life cycle: trogloxenes, troglophiles, and troglobites (Figure 1). Some animals accidentally enter caves because they fall in or are transported by water flow. They are called trogloxenes due to their inability to survive in caves, while troglophiles are cave temporary resident animals. Troglophiles freely move in and out of the cave but need to use the Earth’s surface environment for at least one vital function (i.e., reproduction or feeding). For instance, bats use caves as shelters during the day, and exit at night to forage for insects (Măntoiu et al., 2022). Finally, troglobites are animals permanently confined to subterranean environments, with specific physiological and morphological adaptations to cave habitats (Culver and Pipan, 2009).

In general, the cave biota distribution and adaptation depend on underground environmental conditions. Darkness is undoubtedly the hallmark of cave environments and the first ecological driver for autochthonous organisms. The only alternative to sunlight is the chemical energy deriving from the oxidation of inorganic compounds, supplied by chemolithoautotrophic microorganisms (Sogin et al., 2021). However, caves are oligotrophic environments and the chemical energy required to sustain primary production is limited, except in the case of SAS caves (Brad et al., 2021). All other caves are mostly dominated by heterotrophic organisms that consume organic matter, originating from outside the cave or generated inside by autotrophic production, as energy and carbon sources (Hershey and Barton, 2018). Organic nutrients, comprising particulate organic matter (POM), dissolved organic matter (DOM), and volatile organic carbon (VOC), enter the caves in different ways and quantities (Simon, 2019). They are transported via three main mechanisms: water, air, and animals (Culver and Pipan, 2009). Water is the primary nutrient carrier, enabling the transport of nutrients through flowing and percolation (Pronk et al., 2009). Surface streams can flow directly into void environments dragging organic material in the form of debris or large particles (POM), such as leaves and wood (Simon et al., 2007). For DOM transportation, rainwater first infiltrates through the soil and then into the rock microfractures before reaching the cave level (Simon et al., 2007). Wind and airflow inside the caves can contain spores, pollen, microorganisms, and VOCs (Porca et al., 2011). Animals can also transport nutrients through their movements in and outside the cave (e.g., feces, eggs, dead bodies).

Based on environmental parameters (light, temperature, humidity) and nutrient availability, scientists have generally subdivided caves into four zones, namely the photic, twilight, transition, and deep zones (Figure 1; Lee et al., 2012). The photic zone near the cave entrance mostly harbors phototrophic organisms. Flowering plants (*Spermatophyta*) are rarely found in caves, while ferns, mosses, and lichens form the bulk of the plant biomass in the cave entrance (Glime, 2022).

Progressing from the photic zone to the twilight zone, the light gradually fades depending on the daily and seasonal arc-like path of the sun (Behrendt et al., 2020). At this zone, plants can no longer grow due to low light levels and are generally replaced by algae (e.g., diatoms and green algae) (Falasco et al., 2014). This zone is also enriched with microbial species, such as fungi and bacteria, especially *Cyanobacteriota*, which produce *f* and *d* chlorophylls that allow them to absorb near-infrared (700–780 nm wavelength) radiations for oxygenic photosynthesis (Behrendt et al., 2020). Next, the transition zone is characterized by a complete absence of light, though both temperature and humidity still vary according to the climatic conditions of the Earth’s surface and the seasonal changes (Kosznik-Kwaśnicka et al., 2022).

The transition zone is rich in microbial life, and the extension of this area can vary greatly depending on orography, the altitude at which the cave opens, the size and volume of the cave, and the air circulation inside the cave. Finally, the deep zone is characterized by darkness, almost constant temperature, humidity close to saturation (Badino, 2010), and oligotrophy with less than 2 mg/L of total organic carbon (Hershey and Barton, 2018). Due to its extreme conditions for life, this last zone of the cave is mainly colonized by troglobites (Novak et al., 2012), which have evolved various adaptive traits to darkness and oligotrophy, e.g., depigmentation, loss of sight sensory organs, the utmost development of touch sensory organs, lower metabolism, larger and more slender body shape (Culver and Pipan, 2009). Despite the vast knowledge of fauna caves in the deep zone, microorganisms thriving in this hidden part of the Earth remain elusive due to difficult accessibility, scarcity of biological materials, and challenges in culture-dependent and -independent approaches for the detection of cave microorganisms.

Due to their geographic location, temperate caves show remarkable differences compared with tropical caves. The most evident difference is temperature, with temperate caves typically having cooler temperatures, usually around 8–15°C, while the temperature is around 25°C in tropical caves (Mejía-Ortíz et al., 2020). Interestingly, the biodiversity is considerably higher in tropical caves than in temperate caves. This difference is linked to the greater quantity of energy permanently available in tropical caves, e.g., the abundance of guano resulting from bat colonies (Gnaspini and Trajano, 2000). However, most animals that inhabit tropical caves do not exhibit specific adaptations to cave life, different from troglobites that inhabit temperate caves, where climate is more stable and trophic resources are generally scarce (Howarth, 1980).

## 3 Cave microbiology: an overview

Cave microbiology is a growing research field, continuously providing novel insights into the evolution and adaptation of cave microbial inhabitants. Prominent topics in cave microbiology grossly refer to five research areas: the discovery of new species, geo-microbial interactions, microbial diversity assessment, anthropogenic impacts on cave microbiomes, mechanisms of microbial adaptation, and biotechnological potentials of cave microorganisms.

By analyzing research literature, out of 1,226 research papers retrieved from the Scopus database (December 2023) using “cave AND bacteria” and “cave AND microbes” as keywords, 112 described new bacterial species (nov. sp. cave bacteria as keywords) and their adaptive traits to the cave environment. Due to isolation from the Earth’s surface and the selective pressure imposed by diverse habitats, cave microorganisms can accumulate genetic changes that make them distinct from their surface soil counterparts (Griebler et al., 2014).

Historically, culture-based approaches have been the main strategy for studying cave microbial species. For instance, members of the *Streptomyces* genus have been isolated from Altamira Cave and have been shown to precipitate calcium carbonate in laboratory cultures (Groth et al., 1999). By applying novel high-throughput technologies*, Candidatus* Mycobacterium methanotrophicum was isolated from an extremely acidic biofilm growing on the wall of a SAS cave in Romania (van Spanning et al., 2022). This bacterium represents the first member of *Actinomycetota* to show aerobic methanotrophic properties, previously described only in *Pseudomonadota* and *Verrucomicrobiota*. The probability of discovering new microbial species is likely to increase when different types of caves, or different cave zones, especially the very deep ones, will be explored. Another critical feature of cave microorganisms is their ability to interact with the rock surface (Jones and Northup, 2021). They can contribute to rock dissolution by releasing corrosive organic acids, e.g., oxalic acid and formic acid (Bin et al., 2008), or ligands such as metal-complexing agents, like siderophores, enabling the mobilization of mineral elements necessary for their nutrition (Duncan et al., 2021). The metabolic activity of cave microorganisms can also induce biomineralization or mineral depositions, thereby contributing to the formation of secondary mineral deposits called speleothems (Northup and Lavoie, 2001). For instance, the moonmilk formation is characterized by microcrystalline aggregates textures (i.e., calcite, aragonite, hydromagnesite) resulting from the precipitation of calcite fibers induced by certain cave bacteria, e.g., *Actinomycetota* (Cañaveras et al., 2006; Cacchio et al., 2014; Maciejewska et al., 2017). These biomineralization abilities could be useful in formulating novel bacterial-based building materials (Kosznik-Kwaśnicka et al., 2022).

Furthermore, with the advent of NGS technologies, cave microbial diversity has more intensively been investigated (Tomczyk-Żak and Zielenkiewicz, 2016). Metataxonomic profiling of the prokaryotic hypervariable regions (e.g., V3-V4) of the 16S rRNA gene, the fungal internal transcribed spacer (ITS) rRNA gene, and the eukaryotic 18S rRNA gene unraveled the great diversity of cave microbial communities, which are consortia composed of many species from multiple phyla (Hershey and Barton, 2018). With shotgun sequencing methodologies, the abundance of underexplored domains, including *Archaea*, *Eukarya*, and *Viruses* was revisited (Rossmassler et al., 2016; Kimble et al., 2018; Wiseschart et al., 2019; Turrini et al., 2020). The high species richness in oligotrophic caves remains challenging to explain (Hershey and Barton, 2018). Some authors attribute this phenomenon to competitive exclusion (Prescott et al., 2022), while others suggest interspecies interactions to exploit the scarcity of available nutrients (Barton and Jurado, 2007).

In this context, cave microbial communities are model systems for investigating bacterial relationships and even cell-to-cell communication (Ma et al., 2021). However, comparative studies on cave microbial diversity are challenging due to various factors, including rock types (e.g., limestone, quartz, basalt, gypsum), environmental matrix (wall rock surface, sediment, groundwater water), and the sampling location inside the cave (photic, twilight, transient, and deep zone). As for other extreme environments, caves have attracted researchers to study the ecological implications of resistance to antibiotics (Bhullar et al., 2012), heavy metals (Hamedi et al., 2019), salinity (Farda et al., 2022a), UV radiation (Snider et al., 2009), radioactivity (Enyedi et al., 2019), and desiccation (Vardeh et al., 2018). For instance, in a remote zone at 400 m depth of a pristine cave in New Mexico, isolated for over 4 million years, bacteria were resistant to many antibiotics used in human medicine (Bhullar et al., 2012). Isolate LC231 belonging to *Paenibacillus* sp. was resistant to 26 of 40 antibiotics tested, including daptomycin, a relatively new antibiotic produced by *Streptomyces roseosporus* (Pawlowski et al., 2016). This finding supported the notion that antibiotic resistance mechanisms not only spread in the environment but have existed long before the selection pressure created by human use of antibiotics (Bhullar et al., 2012).

Therefore, caves constitute an excellent model to study the origins and the evolution of the mechanism of resistance in natural microbiomes, unexposed to exogenous interference. It is also clear that caves are fragile ecosystems, extremely susceptible to anthropogenic impact (Bontemps et al., 2022). For instance, show caves which attract tourists due to their beautiful speleothems or paleolithic paintings, alter their autochthonous microbial community structures due to the introduction of allochthonous species of presumptive human origin (Bontemps et al., 2022). The introduction of artificial light sources has been found to induce a progressive proliferation of greenish biofilms named “lampenflora” (Kosznik-Kwaśnicka et al., 2022). Additional factors, including a large number of visitors, and fluctuations in both environmental parameters and nutritional levels, were also associated with microbial community shifts and the appearance of alien species (Alonso et al., 2019; Rachid and Güngör, 2022). However, a dearth of information is currently available about the human impact on other types of caves, including polluted natural caves, especially those in industrial and urban areas (Qian et al., 2020; Scharping and Garey, 2021). Regarding the biotechnological potential, cave autochthonous microorganisms have extensively been investigated for their capacity to produce bioactive compounds (Ghosh et al., 2017a; Rangseekaew and Pathom-aree, 2019; Farda et al., 2022a; Zada et al., 2022). *Actinomycetota*, the main group of antibiotic-producing microorganisms (Barka et al., 2016), are generally abundant in limestone and some volcanic caves (Riquelme et al., 2017; Covington et al., 2018), and interesting antimicrobial properties have been documented for culturable *Streptomyces* strains isolated from moonmilk cave deposits (Maciejewska et al., 2016). Production of novel add-value secondary metabolites has been documented in cave isolates, including cervimycins by *Streptomyces tendae* in a limestone cave from Grotta dei Cervi, Italy (Herold et al., 2005), xiakemycin and huanglongmycin by *Streptomyces* sp. CC8-201 from karst caves in China (Jiang et al., 2018), hypogeamicins by *Nonomuraea* species from Hardin’s cave system in Tennessee, USA (Derewacz et al., 2014), and curamycin by *Streptomyces benahoarensis* from a lava tube in La Palma Island (Canary Islands), Spain (Gonzalez-Pimentel et al., 2022). Recently, *Cyanobacterota* isolates from the lightened walls of the Stiffe cave in Italy were found to produce poly-β hydroxybutyrate for potential use in bioplastics production (Djebaili et al., 2022). Given the problematic culturability of cave bacteria, attempts should be made to implement culture-based strategies for better exploiting their biotechnological potential.

## 4 Main cave systems and microbial colonization patterns

Most studies on cave microbiology have been conducted in limestone, SAS, and volcanic caves. Considering the crucial influence of the mineral matrix on the colonizing microbial communities, hereafter we summarize the mechanisms of formation and the geological structure of limestone, SAS, and lava tubes among volcanic caves, and provide an overview of their microbial diversity.

### 4.1 Limestone caves

**FIGURE 2:**
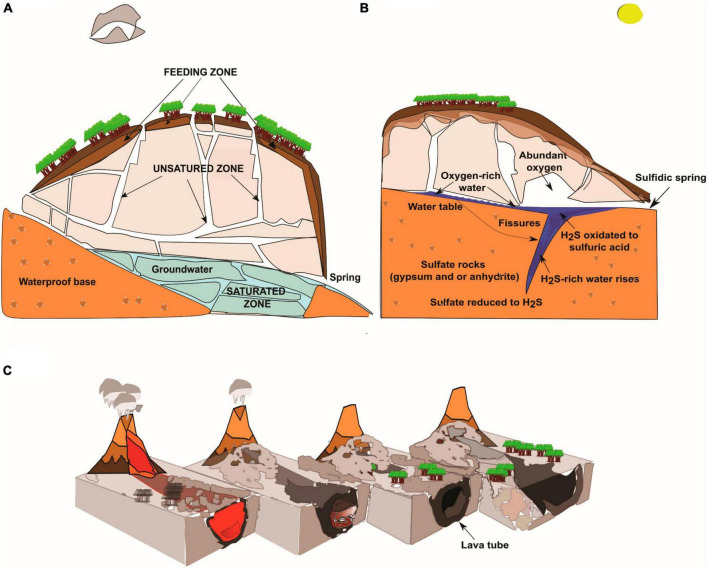
Geological characteristics of limestone, SAS, and volcanic (lava tubes) caves. **(A)** Schematic drawing of an epigenic limestone cave with the three zones of groundwater circulation. The “feeding zone” is the interface zone between the surface and caves that absorbs and collects rainwater and surface runoff waters. The “unsaturated zone” is where superficial waters flow in depth through mainly vertical paths, including wells and meanders or in correspondence with rock fractures. The “saturated zone” corresponds to totally submerged tunnels, ducts, and fractures, where the pressurized waters move in a sub-horizontal direction toward the emergency zone (spring). **(B)** Speleogenesis of hypogenic sulfuric acid caves. Ascending water from the depths of the Earth rich in aggressive substances (H_2_S, CO_2_) induces the dissolution of carbonate rock. **(C)** Formation of lava tubes is the consequence of cooling, emptying, and crusting in the lava flow channel.

Limestone caves are natural cavities in carbonate rocks formed underneath the Earth’s surface, and they can be very different from each other. Some are small, which humans can hardly penetrate, others develop complex networks, which propagate underground for up to several hundred km, reaching over one km in depth (Klusaitė et al., 2016). Most of the largest limestone caves are complex underground structures consisting of rooms, wells, meanders, and intercommunicating tunnels, which are organized to form a system or karst complex (Figure 2A). Mammoth Cave National Park, a World Heritage site in Kentucky, in the USA, has the world’s most extended natural cave network with more than 675 km of surveyed passages. Another World Heritage site, Mulu National Park in Sarawak (Malaysia), contains the world’s largest underground room - Sarawak Chamber in Nasib Bagus Cave. It covers an area of about 160,000 m^2^ and has a volume of about 10 million m^3^. The deepest limestone cave in the world is Verëvkina (Veryovkina) Cave in the Arabika Massif in Abkhazia (Georgia), which is 2,2 km deep (Gulden, 2019).

Dissolution of carbonate rocks requires acidic water that increases the solubility of calcite. CO_2_ present in the atmosphere and soil reacts with rainwater by forming carbonic acid (H_2_CO_3_) (Eq. 1), which further dissociates, producing protons (H^+^) that acidify the solution (Eq. 2). Acid water induces the dissolution of carbonate bedrock to release bicarbonate (HCO_3_^–^) and calcium (Ca^2+^) ions (Eq. 3) (Ford and Williams, 2007). These simple chemical reactions can be expressed as follows:


(1) $${\mathrm{CO}}_{2}+H_{2}\,O↔H_{2}\,{\mathrm{CO}}_{3}$$

(2) $$H_{2}\,{\mathrm{CO}}_{3}↔H^{+}+{\mathrm{HCO}}_{3}^{-}$$

(3) $${\mathrm{Ca}}^{2+}+2\,H\,C\,O_{3}^{-}↔{\mathrm{CaCO}}_{3}+{\mathrm{CO}}_{2}+H_{2}\,O$$

Increasing CO_2_ or decreasing Ca^2+^ drives the Eq. 3 reaction to the left, inducing calcite dissolution, while increasing Ca^2+^ or decreasing CO_2_ drives the Eq. 3 reaction to the right, precipitating calcite.

Irrespective of whether they are epigenic or hypogenic, limestone caves originate from the action of acidic water that flows through the fractures of the bedrock, thus generating subsurface voids. In epigenic caves (Figure 2A), water flows by gravity through the limestone massif causing subterranean drainage (groundwater circulation), and ultimately it returns to the surface at the springs. Conversely, in hypogenic caves, acidic water ascends from the depths of the Earth causing mineral dissolution, as exemplified in SAS caves (Figure 2B and the following session 3.2). A karst system can identify three diverse zones with very different groundwater circulations: feeding, unsaturated, and saturated (Palmer, 1991; Culver and Pipan, 2009; Figure 2A).

**FIGURE 3:**
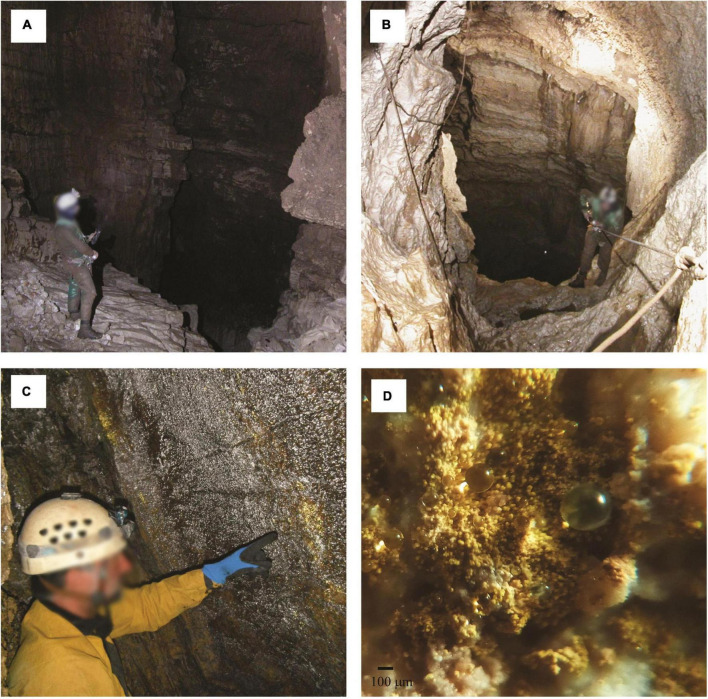
Examples of the unsaturated or vadose zone in limestone caves from Lazio region, Italy. Water is transferred in the deep zone of the cave across vertical wells **(A–B)**. Biofilms colonize the walls near the cave entrance and appear overlaid by water droplets reflecting gold- or silver-colored light when illuminated with an LED lamp **(C)**. Close-up image **(D)** showing yellow microbial communities organized into similarly sized spheroidal structures (about 60 µm in diameter) retaining water droplets due to the condensation of the water vapor. Scale bar (**C**, 100 µm). Photo by P. Turrini.

The lowest zone of the karst system is an extreme oligotrophic environment made of tunnels filled with water (saturated zone) and inhabited by obligate subterranean aquatic animals (stygobionts) whose sustenance depends on chemolithoautotrophic organic matter production (Hutchins et al., 2016). The karst system is not an isolated environment; it is indeed an open system in continuous contact with the external environments by exchanging flows of matter (mainly air, water, and solutes) (Figures 3A, B) and energy (heat) (Waring et al., 2017).

Near the cave entrance, microbial mats overlaid by water droplets, reflecting yellow-gold or silver colored light when illuminated with an LED lamp, can be observed on the walls and ceilings (Pašić et al., 2009; Turrini et al., 2020; Martin-Pozas et al., 2023). These microbial communities are typical of limestone caves and are located predominantly in the trophic transition zone (Porca et al., 2012). Air entering the cave undergoes a decrease in temperature and an increase in relative humidity until it reaches the dew point (Mulec et al., 2015). The water vapor passes to the liquid phase, creating condensation droplets on the surface of the microbial matter (Figures 3C, D). These biofilm-like structures deserve more thorough studies to better understand the role played by water condensation droplets in their maintenance and functionality.

### 4.2 Sulfuric acid speleogenetic (SAS) caves

Some limestone caves originate from carbonate rock dissolution by H_2_S- and H_2_SO_4_-rich water, and therefore they are called SAS caves (Rossmassler et al., 2012; Gulecal-Pektas and Temel, 2017; de Bruin et al., 2022). Although both biotic and abiotic processes are implicated in the development of large sulfuric-acid karsts (Laurent et al., 2023), SAS caves are typically considered hypogenic since they originate from the action of water ascending from the depths of the Earth (Forti et al., 2002). This water contains high concentrations of aggressive substances, primarily H_2_S, and rises through rock fissures and tectonic over-pressuring or hydraulic gradients from sediment basins of the volcanic source to the surface (Figure 2B). When H_2_S-containing fluids get in touch with carbonate bedrock, a cave occurs (Egemeier, 1981). Indeed, H_2_S-rich water meets oxygenated groundwater fed by meteoric infiltration or oxygen from the atmosphere and oxidizes to sulfuric acid (Eq. 4) that immediately reacts with carbonate bedrock by forming gypsum (CaSO_4_ ⋅ 2H_2_O) and CO_2_ (Eq. 5).


(4) $$H_{2}\,S+2\,O_{2}\to H_{2}\,{\mathrm{SO}}_{4}$$

(5) $$H_{2}\,{\mathrm{SO}}_{4}+{\mathrm{CaCO}}_{3}+H_{2}\,O\to {\mathrm{CaSO}}_{4}\cdot 2\,H_{2}\,O+{\mathrm{CO}}_{2}$$

**FIGURE 4:**
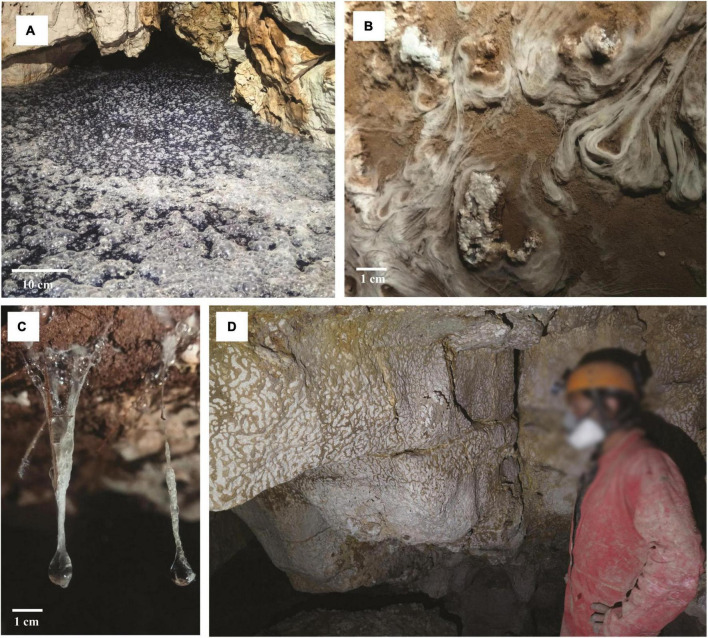
Microbial colonization patterns from a SAS cave in the Lazio region, Italy. Sulfur-oxidizing microbial communities floating on the water surface **(A)** or forming streamers and filaments in the subaqueous environment **(B)**. Microbial communities form highly acidic viscous peduncles called snottites **(C)** or vermiculations on the surface wall rock **(D)**. Scale bar (**A**, 10 cm; **B**, 1 cm; **C**, 1 cm). Photos in **(A)** and **(D)** are by P. Turrini, **(B)** and **(C)** are courtesy of A. Benassi (June 2021).

Gypsum (CaSO_4_ ⋅ 2H_2_O) removal through flowing meteoric water increases void spaces, enlarging the cave (Palmer, 1991; Palmer and Hill, 2019). SAS caves have been reported from many areas worldwide and occur in carbonate rocks in different climates, representing less than 10% of all known caves (Klimchouk, 2017). H_2_S and elemental sulfur are toxic to most organisms, though some chemolithoautotrophic sulfur-oxidizing bacteria can use reduced sulfur compounds as energy sources and electron donors for their energetic metabolisms (Engel, 2007; Kumaresan et al., 2014). In correspondence with the H_2_S-rich rising water, chemolithoautotrophic floating filaments grow on the water surface (Figure 4A; Rohwerder et al., 2003; D’Angeli et al., 2019b), while biofilms thrive as streamers and sedimented filaments in the subaqueous environment (Engel et al., 2003; Jones et al., 2010; Figure 4B). Droplets of freshwater on the walls and ceilings of SAS caves absorb H_2_S degassing from the water table, and chemolithotrophic oxidation of H_2_S to sulfuric acid occurs (Hose et al., 2000). The most acidic drops accumulate on the tips of the gypsum crystals, forming characteristic highly acidic viscous peduncles called snottites (Figure 4C; Hose et al., 2000; Macalady et al., 2006; Jones et al., 2012). These extremely acidic (pH∼1) biofilms attached to cave walls or ceilings are sulfur-based chemosynthetic ecosystems and are observed where H_2_S concentration in the cave atmosphere is 0.2–25 parts-per-million by volume (ppmv) (Jones et al., 2016).

Biovermiculations are typical formations that can be found in SAS caves, located on the surface wall rock at a certain distance from the SAS degassing water. They incorporate microbial populations forming characteristic microbial colonization patterns on the rock resembling leopard skin and appear as worm-like deposits of mud and clay (Figure 4D; Hose et al., 2000; Jones et al., 2008; D’Angeli et al., 2019b). Vermiculations from the Frasassi Cave were found to be composed of densely packed prokaryotic and fungal cells in a mineral-organic matrix containing 5–25% organic carbon (Jones et al., 2008).

### 4.3 Volcanic caves

**FIGURE 5:**
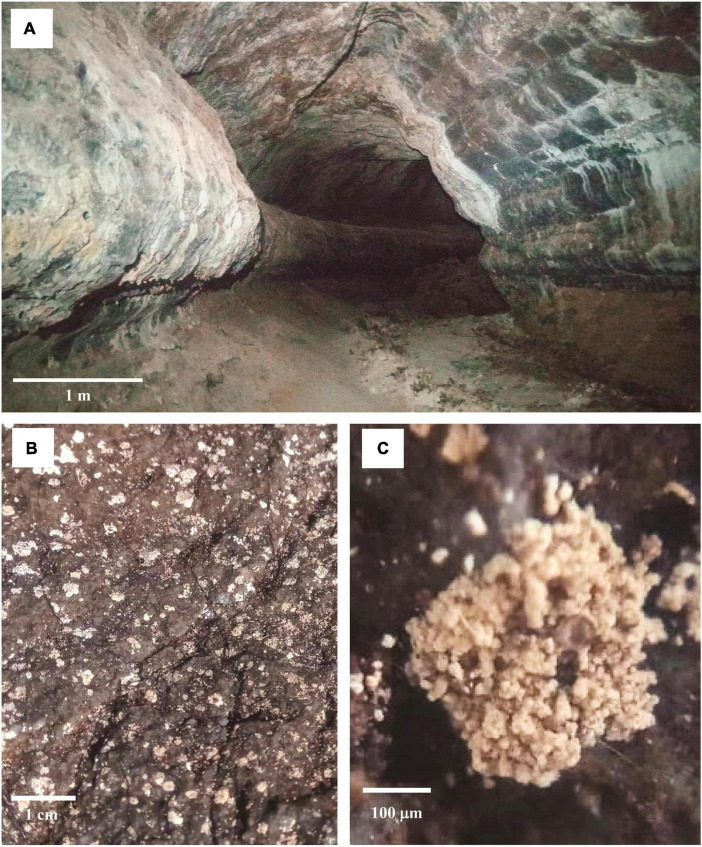
Example of a lava tube. Entrance of Montana Corona cave, Lanzarote, Canary Islands **(A)**. Microbial communities live on the basaltic rock surface **(B)**. Close-up of the microbial communities **(C)**. Scale bar (**A**, 1 m; **B**, 1 cm; **C**, 100 µm). Courtesy of A. Benassi (January 2022).

Different from dissolution caves, volcanic caves are formed following eruption and lava outflow. Molten rock (magma) rises to the surface from depths and loses gas and aqueous vapor forming the so-called lava on the surface. The viscosity of the lava depends on its silica content. Low-silica basalt lava has a low viscosity and can form fast-moving narrow lava streams. Volcanic caves are indeed a large category of cavities in volcanic rocks, and they can be either primary or secondary caves, depending on their genesis (Kempe, 2019). Among primary volcanic caves, lava tubes (Figure 2C) are the most common caves (Palmer, 2007). They are formed as the consequence of cooling and crusting over the lava flow channel, followed by the emptying out of the molten lava leaving behind an empty conduit tube (Figures 2C, 5A; Palmer, 2007; Northup and Lavoie, 2015). Lava tubes are aphotic, shallow subsurface voids that, depending on age and lava texture, can be colonized by plant roots growing through the caves’ ceilings in search of water (Palmer, 2007). These roots, along with water percolating from the surface, bring carbon and nitrogen into lava tubes, creating a mosaic of nutrient availability for diverse microbial mats, mainly consisting of heterotrophic bacteria and fungi, covering the cave walls and ceilings (Figures 5B, C; Hathaway et al., 2014; Riquelme et al., 2015; Gonzalez-Pimentel et al., 2018; Nicolosi et al., 2023). Volcanic caves are widespread worldwide (Gulden, 2019), and contain many secondary mineral deposits with rich biological components that have gained interest as biosignatures for life, aiding the search for life on Mars (Northup et al., 2011). Microbial colonization patterns are visible in a range of colors and shades, including white, yellow, orange, blue-green, gold, and pink mats that cover the walls of lava tubes in various volcanic locations such as the Azores, Hawaii (Hathaway et al., 2014), California (Lavoie et al., 2017), and New Mexico (Northup et al., 2011). These microbial formations are closely associated with secondary mineral deposits, including amorphous copper-silicate deposits and iron-oxide formations (Northup et al., 2011). Coloring is likely to originate from pigments associated with some bacteria present in the colonization patterns, particularly *Actinomycetota* (Lavoie et al., 2017; Gonzalez-Pimentel et al., 2018).

## 5 Microbial diversity in caves

### 5.1 Bacteria in caves

An extensive literature search was conducted to identify the most prevalent bacterial taxa in limestone, volcanic, and SAS caves. Out of 225 research articles screened in our literature analysis, only 20 of them, reporting metataxonomic data for 105 samples, met the inclusion criteria for a comparative assessment of bacterial diversity in caves, as determined by sequence analysis of the V3-V4 hypervariable regions of 16S rRNA using the Illumina (MiSeq) platform (Supplementary Figure 1). With the improvement of deep sequencing technologies, it became possible to detect extremely rare and still unclassified taxa. These are represented by taxonomic units with very low abundance together with unassigned sequences that could not be classified using currently available sequence taxonomy reference databases. Data on cave type, geographic location, and relative distribution of major bacterial taxa in the scrutinized cave samples are provided in Supplementary Dataset 1.

**FIGURE 6:**
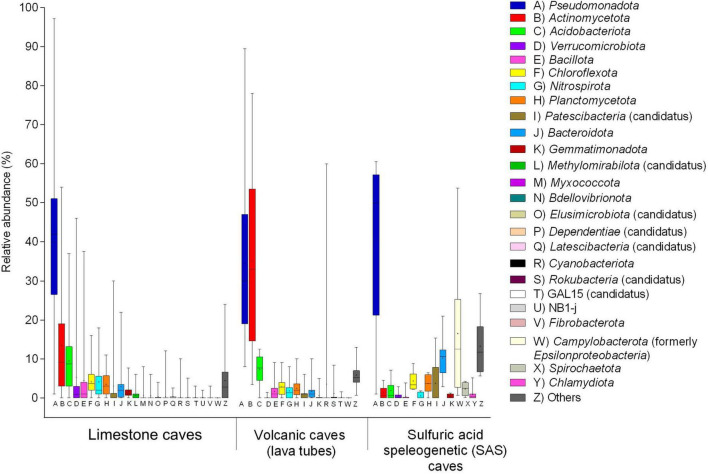
Boxplot of bacterial communities in limestone, volcanic (lava tubes), and SAS caves determined by 16S rRNA gene (V3-V4) sequencing on Illumina platform. Major phyla of pooled samples (water, sediment, biofilm) from various limestone caves retrieved from Leuko et al. (2017), Oliveira et al. (2017), Dhami et al. (2018), Alonso et al. (2019), De Kumar et al. (2019), Long et al. (2019), Dong et al. (2020), Jurado et al. (2020), Park et al. (2020), Addesso et al. (2021), Chen et al. (2021), Koner et al. (2021), Farda et al. (2022b), Bogdan et al. (2023), Martin-Pozas et al. (2023). The phyla pattern of microbial mats on the rock surface of volcanic caves (lava tubes) was obtained from Gonzalez-Pimentel et al. (2018), Gonzalez-Pimentel et al. (2021), Nicolosi et al. (2023). The phyla pattern of biofilms thriving as streamers and sedimented filaments in the water of SAS caves was obtained from D’Angeli et al. (2019a), Jurado et al. (2021). The boundaries for the first and third quartiles are shown (box length), with the centerline representing the median, the symbol (†) indicating the average, and the whiskers representing the maximum/minimum values. Raw data are provided in Supplementary Dataset 1.

Twenty-three prevalent phyla were observed in limestone caves, with major variation in abundance depending on the sampled site and type of matrix. Based on average abundance values of 78 environmental samples from 17 limestone caves worldwide, it can be observed that *Pseudomonadota* were the most abundant phylum (40.0%), followed by *Actinomycetota* (13.5%), *Acidobacteriota* (9.5%), *Verrucomicrobiota* (5.3%), *Bacillota* (4.5%), *Chloroflexota* (4.1%), *Nitrospirota* (4.1%), *Planctomicetota* (3.5%), *Candidatus* Patescibacteria (3.0%), *Bacteroidota* (2.9%), *Gemmatimonadota* (1.7%), *Candidatus* Methylomirabilota (0.7%), *Myxococcota* (0.6%), *Bdellovibrionota* (0.5%), *Candidatus* Elusimicrobiota (0.4%), *Candidatus* Dependentiae (0.3%), *Candidatus* Latescibacteria (0.3%), *Cyanobacteriota* (0.2%), *Candidatus* Rokubacteria (0.2%), *Candidatus* GAL15 (0.1%), NB1-j (0.1%), *Fibrobacterota* (>0.1%), *Campylobacterota* (>0.1%), and others (4.5%) (Figure 6). The overall distribution of prevalent phyla was retained irrespective of the geographical location of the caves, and was remarkably similar to that of volcanic caves (lava tubes), even if *Actinomycetota* were more abundant in the latter cave type (Figure 6). Based on average abundance values of 17 environmental samples from 3 lava tubes worldwide, a predominance of *Actinomycetota* (35.0%) and *Pseudomonadota* (34.6%) was observed, followed by *Acidobacteriota* (7.4%), *Cyanobacteriota* (3.5%), *Chloroflexota* (2.8%), *Planctomycetota* (2.5%), *Nitrospirota* (1.8%), *Bacteroidota* (1.7%), *Bacillota* (1.7%), *Candidatus* Rokubacteria (1.2%), *Candidatus* Patescibacteria (1.1%), *Gemmatimonadota* (0.7%), *Verrucomicrobiota* (0.2%) and others (5.9%) (Figure 6).

The average phyla distribution in 10 environmental samples from two SAS caves showed the dominance of *Pseudomonadota* (40.0%) and *Campylobacterota* (16.5%), followed by *Bacteroidota* (10.2%), *Chloroflexota* (4.4%), *Candidatus* Patescibacteria (3.8%), *Planctomicetota* (3.7%), *Spirochaetota* (2.3%), *Acidobacteriota* (1.9%), *Actinomycetota* (1.2%), *Chlamydiota* (0.9%), *Verrucomicrobiota* (0.6%), *Nitrospirota* (0.5%), *Bacillota* (0.5%), *Gemmatimonadota* (0.4%) and others (13.3%) (Figure 6).

**FIGURE 7:**
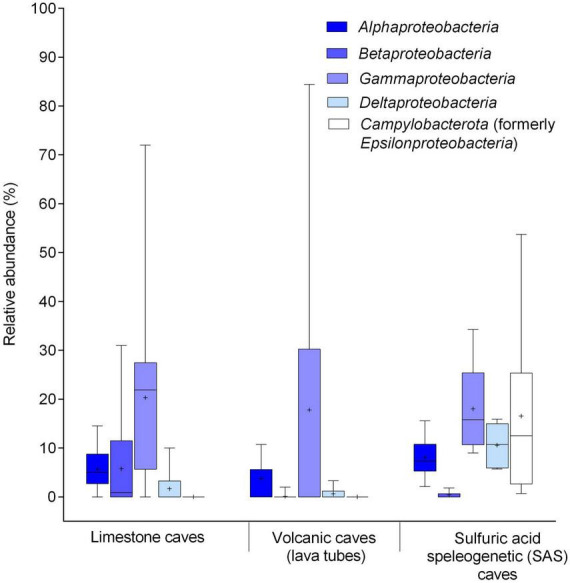
Boxplot of bacterial communities in limestone, volcanic (lava tube), and SAS caves determined by 16S rRNA gene (V3-V4) sequencing on Illumina platform. Relative composition of *Pseudomonadota* at the class level and *Campylobacterota* (heterotypic synonym *Epsilonproteobacteria*) phylum. Major classes of pooled samples (water, sediment, biofilm) from various limestone caves retrieved from Leuko et al. (2017), Oliveira et al. (2017), De Kumar et al. (2019), Jurado et al. (2020), Park et al. (2020), Addesso et al. (2021), Farda et al. (2022b), Martin-Pozas et al. (2023). The class pattern of microbial mats from volcanic caves (lava tubes) was obtained from Gonzalez-Pimentel et al. (2018), Gonzalez-Pimentel et al. (2021), Nicolosi et al. (2023). The class pattern of biofilms thriving as streamers and sedimented filaments in the water of SAS caves was obtained from D’Angeli et al. (2019a), Jurado et al. (2021). The boundaries for the first and third quartiles are shown (box length), with the centerline representing the median, the symbol (†) indicating the average, and the whiskers representing the maximum/minimum values. Raw data are provided in Supplementary Dataset 1.

Following the updated taxonomic revision of bacterial phyla (Oren and Garrity, 2021), the class-level distribution of the *Pseudomonadota* members (*Alpha*-, *Beta*-, *Gamma*-, and *Delta-proteobacteria*) and the *Campylobacterota* phylum (formerly *Epsilonproteobacteria*) were compared for the three types of caves. A remarkably different distribution of taxa was observed in the three cave types; *Gammaproteobacteria* predominated in limestone and volcanic caves, whereas the phylum *Campylobacterota* (heterotypic synonym *Epsilonproteobacteria*) dominated in SAS caves (Figure 7). In addition to the Fetida Cave, Italy (Figure 7), also the Lower Kane Cave, USA (Engel et al., 2003), the Frasassi Cave, Italy (Macalady et al., 2008; Zerkle et al., 2016) and the Acquasanta Terme Cave, Italy (Jones et al., 2010) showed *Campylobacterota* as the major contributor to microbial mats.

According to the selected literature (Supplementary Dataset 1), the taxonomic data of bacterial communities from volcanic (lava tubes) and SAS caves are mainly referred to biofilms and microbial aggregates collected from the rock surfaces and H_2_S-rich groundwater, respectively (Figure 6 and Supplementary Dataset 1).

**FIGURE 8:**
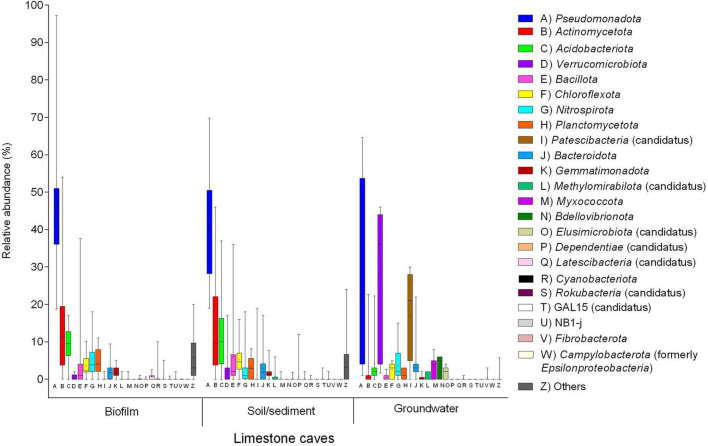
Distribution of the main bacterial phyla in three types of matrices (water, biofilm from wall surfaces, and sediment) from limestone caves determined by 16S rRNA gene (V3-V4) sequencing on Illumina platform. Phyla distributions of the biofilm samples were obtained from Leuko et al. (2017), Alonso et al. (2019), De Kumar et al. (2019), Long et al. (2019), Dong et al. (2020), Jurado et al. (2020), Addesso et al. (2021), Chen et al. (2021), Koner et al. (2021), Martin-Pozas et al. (2023). Phyla distributions of the sediment samples were obtained from Oliveira et al. (2017), Dhami et al. (2018), Dong et al. (2020), Park et al. (2020), Koner et al. (2021), Farda et al. (2022b), Bogdan et al. (2023), Martin-Pozas et al. (2023). Phyla distribution of the water samples was obtained from Chen et al. (2021), Bogdan et al. (2023), Martin-Pozas et al. (2023). The boundaries for the first and third quartiles are shown (box length), with the centerline representing the median, the symbol (†) indicating the average, and the whiskers representing the maximum/minimum values. Raw data are provided in Supplementary Dataset 1.

Three different types of matrices, namely biofilms, sediments, and water, are commonly investigated at the microbiological level in limestone caves. Figure 8 illustrates pooled data on the relative abundance of bacterial phyla in biofilm, sediment, and groundwater samples (33, 34 and 11 samples, respectively) taken from 17 limestone caves worldwide. The most abundant phyla in biofilm and sediment samples were (respectively) *Pseudomonadota* (45.1–40.9%) followed by *Actinomycetota* (15.0–15.3%), *Acidobacteriota* (9.0–11.6%), *Verrucomicrobiota* (0.6–2.4%), *Bacillota* (4.0–6.1%), *Chloroflexota* (3.6–5.2%), *Nitrospirota* (5.0–3.0%), *Planctomycetota* (4.6–3.1%), *Bacteroidota* (2.3–2.9%), *Gemmatimonadota* (1.9–2.0%), and others (5.7–4.3%). Differently, the abundance of phyla in water samples was *Verrucomicrobiota* (28.3%)*, Pseudomonadota* (22.3%), *Candidatus* Patescibacteria (16.9%), *Bacteroidota* (4.4%), *Acidobacteriota* (4.2%), *Nitrospirota* (3.7%), *Myxococcota* (3.5%), *Bdellovibrionota* (3.5%), *Actinomycetota* (3.4%), *Chloroflexota* (2.5%), *Candidatus* Elusimicrobiota (2.1%), *Planctomicetota* (1.5%), *Candidatus* Methylomirabilota (1.2%), *Bacillota* (0.4%), *Fibrobacterota* (0.4%), *Gemmatimonadota* (0.3%), *Cyanobacteriota* (0.2%), and others (1.0%).

It was interesting to notice the very similar distribution of bacterial phyla in biofilms and sediments, both showing remarkable differences from water samples taken from the same cave. Sediments are likely to serve as a reservoir and source of bacteria which can spread on cave wall surfaces giving rise to biofilm formations on suitable rock matrices under permissive environmental conditions (Martin-Pozas et al., 2023). Overall, one major difference between groundwater samples and both biofilm and sediment samples was the prevalence of *Verrucomicrobiota* and *Candidatus* Patescibacteria, and the relative scarcity of *Actinomycetota*. This is not surprising, given that *Verrucomicrobiota* and *Candidatus* Patescibacteria are prominently aquatic taxa, as opposed to the ubiquitous *Actinomycetota* that can thrive in both terrestrial and aquatic environments (Dhami et al., 2018). Indeed, *Verrucomicrobiota* were detected with high abundance in cave drip water, whereas they were rare in sediments and biofilms (Bogdan et al., 2023; Martin-Pozas et al., 2023).

### 5.2 Functional roles of bacteria in limestone and volcanic caves

The success of *Pseudomonadota* in colonizing cave environments may partly be attributed to their involvement in sulfur cycling, and their ability to degrade a wide range of organic compounds, fix atmospheric carbon, and transform nitrogen (Tomczyk-Żak and Zielenkiewicz, 2016). Among *Gammaproteobacteria*, the prevalent orders in caves are *Pseudomonadales*, *Xanthomonadales*, and *Chromatiales*, which are generally associated with freshwater (Porca et al., 2012; Hershey et al., 2018). In the *Alphaproteobacteria* class, *Rhizobiales* are typically associated with nitrogen fixation (Carvalho et al., 2010), and are generally found near the cave entrance, their tropism being influenced by the nearby rhizosphere community and the availability of nutrients from root exudates (Michail et al., 2021). *Sphingomonadales* can degrade aromatic compounds (Marques et al., 2019). *Caulobacterales* (e.g., members of the *Brevundimonas* genus) are often isolated from caves (Ghosh et al., 2017b; Zhu et al., 2021). Within the class *Betaproteobacteria*, the dominant order is *Nitrosomonadales* (including *Nitrosomonas* and *Nitrosospira*) which are ammonia-oxidizing bacteria (Zhao et al., 2017; Kimble et al., 2018; Jurado et al., 2020). *Actinomycetota* colonize both limestone and volcanic caves, mainly due to their broad adaptive abilities. For instance, they can produce degradative enzymes for numerous natural macromolecules (Riquelme et al., 2017; Hamedi et al., 2019), and release secondary metabolites such as antibiotics to prevent nutrient withholding by competitors under oligotrophic conditions (Barka et al., 2016; Maciejewska et al., 2016). *Crossiella* (*Pseudonocardiales*) is a common inhabitant of both limestone and volcanic caves and is often isolated from moonmilk deposits, where it plays a critical role in calcite precipitation through urease activity-dependent alkalinization (Gonzalez-Pimentel et al., 2021). *Crossiella* also provided a new model of bacterial proliferation and dispersion in caves, since free *Crossiella* cells from surface and underground sediments can attach to cave walls and form the first filaments that subsequently evolve into mature biofilms (Martin-Pozas et al., 2023). The family *Euzebyaceae* has been reported to be abundant in microbial mats of lava tubes (Riquelme et al., 2015; Gonzalez-Pimentel et al., 2018) and limestone caves (Ma et al., 2021).

Bacteria of the genus *Streptomyces* (Maciejewska et al., 2017; Oliveira et al., 2017; Long et al., 2019) are the most abundant producers of antimicrobials and play a role in maintaining the microbial community by inhibiting the growth of surrounding microorganisms (Park et al., 2020). Bacteria belonging to the phylum *Acidobacteriota* are ubiquitous in various terrestrial environments (Kalam et al., 2020) and relatively abundant in caves (Figure 6 and Supplementary Dataset 1). Although acidobacterial sequences are preponderant in soil samples and account for a significant fraction of cave microbiomes, the ecological role and metabolic function(s) of these bacteria remain elusive due to their recalcitrance to laboratory cultivation. Some *Acidobacteriota* members have been classified as k-strategists, in that they have oligotrophic metabolism which enables them to thrive in settings with limited nutritional availability with slow growth rates (Kalam et al., 2020). These features are likely to contribute to the environmental fitness of *Acidobacteriota* in caves (Fierer et al., 2007). *Bacillota* is a phylum frequently found in caves due to their resistance to desiccation and nutrient stress (Dong et al., 2020). Both anaerobic (e.g., *Clostridium* spp.) and aerobic (e.g., *Bacillus* spp.) spore-formers were detected in caves (Oliveira et al., 2017). The *Chloroflexota* are CO_2_-fixing autotrophic green non-sulfur bacteria that frequently colonize volcanic caves, where they represent one of the most abundant phyla (Riquelme et al., 2015).

While nitrogen is a limiting nutrient in caves (Anda et al., 2020), the chemolithoautotrophic members of phylum *Nitrospirota* are relatively abundant and ubiquitous in subterranean environments, where they play a role in primary production by assimilating inorganic carbon (Lücker et al., 2010; Mueller et al., 2023). The ammonia-oxidizing *Nitrosospira* and nitrite-oxidizing *Nitrospira* complete the entire nitrification cycle and support primary production in oligotrophic environments (Lavoie et al., 2017; Leuko et al., 2017). *Nitrospirota* members were detected in a variety of subterranean environments, e.g., on a cave wall in the western Loess Plateau of China (Anda et al., 2020), in the Su Bentu limestone cave in Sardinia (Leuko et al., 2017) and the limestone Pindal Cave in Spain (Martin-Pozas et al., 2023), but also in speleothems of a silicate cave in Guiana (Liu et al., 2022) and in the lava tubes of Lava Beds National Monument, USA (Lavoie et al., 2017). Members of *Thermodesulfovibrionia*, a class of *Nitrospirota* characterized by hydrogen oxidation, sulfate reduction, nitrate reduction, and sulfur disproportionation, are rare in surface environments but are frequent in marine and terrestrial subsurface aquifers (D’Angelo et al., 2023).

*Bacteroidota* are common inhabitants of caves (Figure 6 and Supplementary Dataset 1) and have been proposed as bioindicators of human disturbance since their relative abundance significantly increased in a cave open to tourists (Alonso et al., 2019). Indeed, the most convincing alteration of the microbiota caused by touristic visits to show caves is the occasional introduction of human commensal microorganisms, like *Staphylococcus* spp. or *Enterobacteriaceae* (Espino del Castillo et al., 2018; Bontemps et al., 2022). Seemingly, the overall distribution of microbial phyla in caves reflects what is observed in the soil (Hershey and Barton, 2018). It should be considered that microorganisms can constantly gain access to the caves through entrances, underground streams, air currents, and percolating water, continuously modifying the native cave microbial communities (Wu et al., 2015). However, at lower taxonomic levels, only 16 % overlap of OTUs was observed between the cave and external microbiomes in the limestone Kartchner Caverns, Arizona (Ortiz et al., 2014), and 11.2 % between surface soil and microbial mats collected from lava tubes of Lava Beds National Monument, California (Lavoie et al., 2017). These data support the hypothesis that cave-indigenous microbial populations differ from surface soil populations. Indeed, Earth’s surface microorganisms can migrate into the caves, but once there, environmental, and chemical factors determine a selective pressure that selects those microorganisms that better adapt to the harsh conditions dictated by the caves.

Most cave microbiology studies have been performed in easily accessible shallow caves or near cave entrances, while limited information is available in the deep zone. In the Krubera-Voronja Cave, one of the deepest limestone caves in the world, high microbial diversity was observed at the phylum and genus levels (Kieraite-Aleksandrova et al., 2015). *Pseudomonadota* were most abundant in the lower parts of the cave, while *Actinomycetota* dominated in the upper parts, presumably due to differences in organic carbon availability between these two zones (Kieraite-Aleksandrova et al., 2015). In the case of limestone caves, there is some evidence of the presence of cave-adapted bacteria. Indeed, a core microbiome was observed in the golden droplet-forming microbial communities inhabiting three geographically separate limestone caves, namely Altamira Cave in Spain, Sloup-sosuvka caves in the Czech Republic, and Pajsarjeva Jama in Slovenia (Porca et al., 2012). About 60% of the 16S rRNA full-length gene sequences formed three core OTUs common to all three sampling sites. These were referred to *Pseudonocardinae* (30–50% of sequences), *Chromatiales* (6–25% of sequences), and *Xanthomonadales* (0.5–2.0% of sequences). Interestingly, the bacterial communities inhabiting the rock surfaces in a limestone cave located on the western Loess Plateau of China (Wu et al., 2015) and the moonmilk samples collected from a limestone cave in South Korea (Park et al., 2020) were dominated by some phylotypes showing high similarity with phylotypes identified in geographically distinct European caves (Porca et al., 2012).

Furthermore, a robust core microbiome of shared ASVs (15.1%) between five limestone caves located in different regions of Italy (Biagioli et al., 2023) supported the high degree of adaptation and specialization for microbial communities in limestone caves. Different from limestone caves, studies on volcanic caves from geographically distant areas highlighted a low number of shared OTUs among microbial communities, suggesting that slight differences in lava chemistry or other microenvironmental factors may affect the microbial community structure of volcanic caves (Hathaway et al., 2014; Gonzalez-Pimentel et al., 2018; Prescott et al., 2022). In particular, differences in mineral composition and the high porosity of volcanic rocks could favor the diversification of the endemic microbial communities. In this context, the microbial community can vary because of the interaction with different mineral components of volcanic rocks (Jones and Bennett, 2014). In contrast, limestone caves comprise less porous and mineral-poor carbonate rocks. This makes limestone caves more similar to each other, even if located in geographically distant karst areas. The uniform nature of the carbonate rock could therefore select specific taxa, well adapted to limestone subterranean substrates.

### 5.3 Functional roles of bacteria in SAS caves

*Campylobacterota* (Waite et al., 2017), are the most abundant bacteria in SAS caves (Figure 7; Engel et al., 2003; Macalady et al., 2006). Members of *Campylobacterota* are prominently associated with sulfur metabolism, causing both sulfide oxidation and sulfate reduction, thereby completing the sulfur cycle (Barton and Luiszer, 2005). Microbial communities associated with SAS caves include stream biofilms, snottites, and biovermiculations (Figure 4). In correspondence with the H_2_S-rich rising water, chemolithoautotrophic biofilms thrive as streamers and sedimented filaments in the subaqueous environment (Engel et al., 2003, 2004a; Macalady et al., 2006; Jones et al., 2010), while floating filaments grow on the water surface (Rohwerder et al., 2003; D’Angeli et al., 2019b). Studies on water streamer communities in Lower Kane Cave and Frasassi Cave have confirmed the prevalence of sulfur-oxidizing microorganisms, particularly *Campylobacterota* which dominate in waters with high H_2_S and low oxygen, while *Thiothrix* (*Thiotrichales, Gammaproteobacteria*) dominate in waters with low H_2_S and high oxygen levels (Engel et al., 2004a; Macalady et al., 2008). *Beggiatoa* (*Thiotrichales, Gammaproteobacteria*) was the dominant group in locations where the shear stress caused by the flowing water is low enough to allow fine sediment accumulation, regardless of sulfide and oxygen concentration (Macalady et al., 2008). In the sulfidic spring of Fetida Cave, Italy, the microbial biomass of water filaments was dominated by the genus *Arcobacter* within the phylum *Campylobacterota* (Jurado et al., 2021). Notably, *Arcobacte*r species are primary producers because capable of fixing CO_2_ and growing chemolithotrophically via sulfur oxidation. *Desulfocapsa* (Jurado et al., 2021) are anaerobic sulfate-reducing bacteria that produce H_2_S useful for sulfur-oxidizing bacteria (Macalady et al., 2006, 2008; Keeler and Lusk, 2021). Snottites are extremely acidic (pH∼1) biofilms growing on the walls and ceilings of caves where sulfide-rich springs degas H_2_S into the cave atmosphere (Figure 4C; Jones et al., 2012). In Frasassi Cave, snottites are dominated by *Acidithiobacillus* (*Gammaproteobacteria*, 71.4–75.4%), *Thermoplasmatales* (15.6–20.0%), and *Acidimicrobiaceae* (*Actinomycetota*, 5.7–7.0%), in addition to several low-abundance taxa (Macalady et al., 2007). *Acidithiobacillus* are sulfide-oxidizing chemolithoautotrophic bacteria, while *Acidimicrobiaceae* and *Thermoplasmatales* are capable of organotrophic or mixotrophic growth via sulfide and organic carbon oxidation (Macalady et al., 2007).

Biovermiculations are organic-rich sediment formations, showing a typical deposition pattern, found on the walls of some SAS caves (Figure 4D). The diversity of biovermiculation patterns has been studied in Fetida Cave and Frasassi Cave, both located in Italy (Jones et al., 2008; D’Angeli et al., 2019b), and Cueva de Villa Luz Cave in Mexico (D’Auria et al., 2018). Biovermiculations are mainly composed of bacteria belonging to the *Betaproteobacteria*, *Gammaproteobacteria*, *Acidobacteriota*, *Nitrospirota*, and *Planctomycetetota*. Members of *Hydrogenophilales* (*Hydrogenophilia*) and *Acidiferrobacterales* (*Gammaproteobacteria*) obtain energy from sulfur and iron oxidation, and perform carbon fixation (D’Angeli et al., 2019b). In biovemiculations, the presence of *Nitrospirota* suggests an energetic metabolism based on the oxidation of reduced nitrogen compounds, together with chemoheterotrophy, as inferred by the presence of hydrocarbon-degrading bacteria (*Hydrocarboniphaga*, *Gammaproteobacteria*), and members of *Acidobacteriota* and *Actinmycetota* (Jones et al., 2008).

### 5.4 Fungi in caves

Fungi are important components of cave microbiotas, particularly *Ascomycota* which are the predominant phylum. Genus *Candida* (class *Saccharomycetes*) was found abundant in caves open to tourists where visitors may act as a primary vector of human commensal fungi (Biagioli et al., 2023). Other fungal phyla found in caves are *Basidiomycota, Zygomycota*, and *Mycetozoa* (Vanderwolf et al., 2013; Carmichael et al., 2015). Fungal taxa so far recognized in caves are those commonly found on the Earth’s surface, and air currents entering and circulating within caves can distribute spores from the outside environment. It has also been suggested that the presence of fungi in caves is an indirect consequence of the entry of organic matter vehicled by cave animals, human visitors, and airborne spores from outside (Martin-Pozas et al., 2022). Fungi are not distributed evenly throughout caves, being commonly associated with organic debris and cave fauna. The genus *Mortierella*, a psychrotolerant cellulose-degrading fungus belonging to *Mortierellomycota*, was found particularly abundant in wild natural caves, primarily associated with the macro-fauna components, i.e., rodents and bats (Biagioli et al., 2023). Fungi in caves generally act as decomposers or parasites, and several fungal species in caves are known to parasitize cave insects (Benoit et al., 2004). Mycorrhizal fungi can also be found in association with plant roots that penetrate shallow caves, such as lava tubes.

Bats can be vectors for fungal spores in and out of the cave environments. Bat guano is the most common source of organic matter where several fungal species grow (Vanderwolf et al., 2013). Among these, *Histoplasma capsulatum* is the most widely studied fungus in caves, being the etiological agent of histoplasmosis, a potentially fatal disease acquired by the inhalation of spores vehicle by bats, endemic to Southeast Asia, Australia, Africa, and parts of South and North America (Taylor et al., 2022).

### 5.5 *Archaea* in caves

*Archaea* are generally found in caves, and their abundance in microbial communities seems to be quite small (<2%) (Hershey and Barton, 2018), although their population size can increase especially under oligotrophic conditions (Cheng et al., 2023). *Archaea* play a key role in the nitrogen cycle (Kimble et al., 2018), the predominance of the phylum *Thaumarchaeota* in archaeal cave populations is mainly attributed to its ability to oxidize nitrogen compounds (e.g., ammonia and nitrites) even under low nitrogen conditions, and autotrophically generate organic molecules for growth (Ortiz et al., 2014; Wiseschart et al., 2019). Other archaeal taxa in cave communities are *Euryarchaeota, Crenarchaeota*, and *Woesearchaeota* phyla (Alonso et al., 2019; Biagioli et al., 2023). The role and activity of *Archaea* in cave ecosystems remain enigmatic (Hershey and Barton, 2018; Alonso et al., 2019). Their composition in caves revealed similar patterns as those found on plants, suggesting that some *Archaea* could originate from plants’ rhizosphere growing above caves and transported inside caves by water infiltration (Bontemps et al., 2022). This hypothesis is reasonable for microbial communities thriving near the cave entrances (photic, twilight, and transient zones) where matter transfer from the external environment occurs.

## 6 Good practices in future cave microbiome studies

While most natural and man-made caves are still unexplored from a microbiological perspective, the fascinating idea is gaining ground that caves are not only model ecosystems to investigate microbial adaptation to extreme conditions (pH, temperature, oligotrophy, darkness, presence of potential growth inhibitors, etc.) but also valuable reservoirs of microbial diversity and potential sources of biotechnologically relevant microbial species. Literature analysis has evidenced some methodological heterogeneity in the design and conduction of studies aimed to characterize cave microbial communities, complicating the comparison of results, data processing, and the proposal of prototypical microbiomes for different types of caves and/or environmental samples. Microbiological investigation of caves requires careful planning and standardization of methodological approaches, to make data from different studies comparable. Hereafter we shall briefly discuss some methodological approaches to cave microbiome investigations, encompassing “in-cave”, “in-lab”, and “in-silico” studies.

It is advisable for microbiologists to join regional/international speleological groups to obtain permission and assistance for access to caves. Conventions with the local authorities should be signed whenever caves are in a protected natural area or signatory countries of the Nagoya Protocol agreement (McCluskey et al., 2017).

For in-situ studies, the map, GPS coordinates, and elevation above sea level of the cave entrance should be determined. Sampling sites along the four cave trophic zones (i.e., photic, twilight, transient, and deep zones) should be mapped and reported in data repositories and/or research papers. To define these zone borders, environmental parameters including light intensity (i.e., photosynthetic active radiation PAR, λ = 400–700 nm), temperature (°C), humidity (%), and TOC (mg/L, mg/Kg) should be measured at each sampling site and, preferably, monitored during the time using *in situ* dataloggers (Mejía-Ortíz et al., 2020). The TOC concentration determines the nutrient availability across the cave zones, and it should be determined in the groundwater and sediments. Standard physicochemical characterization of the environmental matrices (e.g., groundwater, rock, air, sediment) would aid in understanding the metabolic activity of microbial inhabitants and the local biogeochemical cycling (D’Angeli et al., 2019b; Zada et al., 2022; Zhu et al., 2022). For instance, portable spectrophotometers and Fourier Transform Infrared (FTIR) spectroscopes can detect the presence of some organic and inorganic compounds (e.g., heavy metals) potentially involved in microbial metabolism (Macalady et al., 2008).

Recent advances in imaging and camera resolutions make it possible to take high-resolution photographs of microbial colonization patterns. Macro-photography can be carried out whether using *in situ* optical microscopy lenses (with appropriate adapters) or employing in-lab optical microscopy on environmental matrix samples. It is recommended to change the focal plane during the picture collection to reconstruct the focused three-dimensional object (e.g., with Helicon focus software). Scanning electron microscopy (SEM) is a powerful tool for imaging and analyzing cave microbiome morphologies, generating images with very high magnifications (He et al., 2021). It can also provide elemental analysis. However, SEM requires *ex-situ* preparation steps (fixation, dehydration, and coating) that can alter the native structure of the sample. Whenever possible, samples should be kept refrigerated (4°C) or frozen (<20°C) during transportation to the laboratory.

For in-lab studies, cell count is also a key parameter to estimate the microbial population size in different cave matrices (e.g., biofilm, air, sediment, and groundwater). Several methods have been proposed for the quantification of bacteria in water (Hershey et al., 2019) and soil (Lee et al., 2021). Fluorescence *in situ* hybridization (FISH) can identify and quantify specific microbial taxa in environmental samples, allowing for direct microscopic observation using epifluorescence or confocal laser scanning microscopies (Jones et al., 2023).

Metagenomic analysis of environmental samples can provide a comprehensive view of the structure and dynamics of microbial communities thriving in caves. To analyze eDNA samples, two approaches could be used. In one case, biological replicates (≥2 from the same cave site and matrix) could be extracted, sequenced, and analyzed separately [e.g., (D’Angeli et al., 2019b)]. Otherwise, samples from multiple homogeneous sites can be collected and pooled before eDNA extraction, to capture the full range of microbial diversity in a specific cave site (Biagioli et al., 2023). Sample pooling increases the quantity and diversity of eDNA and ensures statistical robustness, though it affects the alpha and beta diversity indices and reduces the sensitivity required for the detection of rare taxa (Beng and Corlett, 2020; Weinroth et al., 2022). Following the extraction of eDNA, the microbial community structure can be determined by amplicon metataxonomic profiling (e.g., 16S rRNA, ITS, 18S rRNA) using appropriate primers for specific domains of life (e.g., Archaea, Bacteria, Eucaryotes, Fungi, Viruses), in combination with NGS technologies. Recent portable NGS platforms (e.g., MinION) can apply to cave microbiome studies due to low cost and rapid workflow (Latorre-Pérez et al., 2020; Wang et al., 2021). This approach offers acceptable accuracy when combined with novel algorithms of error corrections and other NGS technologies (e.g., Illumina) (Nygaard et al., 2020; Kovaka et al., 2023). The shotgun metagenomic approach involves the untargeted sequencing of all microbial genomes in the eDNA sample. This approach provides a more accurate taxonomic profile of the entire microbial community and can substantiate metabarcoding results. Recovering whole genome sequences can also provide essential information about the functional properties of the microbial community through the reconstruction of metabolic pathways (Quince et al., 2017; Chiciudean et al., 2022). *De novo* assembly of metagenome samples has been applied to studying cave microbial communities (Rossmassler et al., 2016; Kimble et al., 2018; Wiseschart et al., 2019; Turrini et al., 2020) and constructing microbial genomes, known as MAGs (van Spanning et al., 2022).

MAGs provide comprehensive coverage of the genetic diversity in a microbial community (Asnicar et al., 2020; Kashaf et al., 2021), and predict the involvement of individual components in nutrient cycling (Bendia et al., 2022) and symbiotic relationships (Wang et al., 2022). Metatranscriptomics consists of sequencing and quantifying the relative abundance of mRNAs in a sample to determine the functional activities of a given microbiome (Bashiardes et al., 2016). Although crucial for understanding the regulation of gene expression in microbial communities (Zhang et al., 2021), this technique has occasionally been applied to cave microbiome research (Mondini et al., 2022). It could be combined with proteomics and metabolomics (Idle and Gonzalez, 2007; Roldán et al., 2018) to systematically quantify protein expression and identify metabolites from cave samples. It is worth noting that despite the great number of metataxonomic profiling studies from caves, only a few of them have employed high throughput metabolite characterization on pure bacterial strains from caves (Gosse et al., 2019; Zhu et al., 2021).

Culture-dependent approaches make it possible to obtain pure cultures of cave microorganisms and to gain insights into the role played by individual components of the community. However, only a very low fraction, as less as ca. 1% of cave bacteria, will grow on standard laboratory media (Bodor et al., 2020). To increase the recovery of microbial species, one possibility is to move cultivation from the lab into the cave environment. To this purpose, a diffusion chamber (called “isolation chip” or “ichip”) in which cells from environmental samples are diluted in agar and posed between two membranes with 20- to 30-nm pore size, can be used. Once the chamber is returned to the original environment (e.g., water, sediment), naturally occurring nutrients and growth factors diffuse into the chamber, fulfilling the growth requirements of individual components of the microbial community, ultimately increasing their recovery rate. In contrast, the movement of bacteria into/out of the chamber is prevented (Nichols et al., 2010). Microcosm experiments can also provide useful information on the ecology of cave microorganisms, as they allow systematic manipulation of environmental parameters to examine their impact on microbial communities (Vos et al., 2013). Enrichment cultures using definite nutrients and energy sources can also allow the selection of taxa endowed with specific metabolic properties from the microbial pool.

**FIGURE 9:**
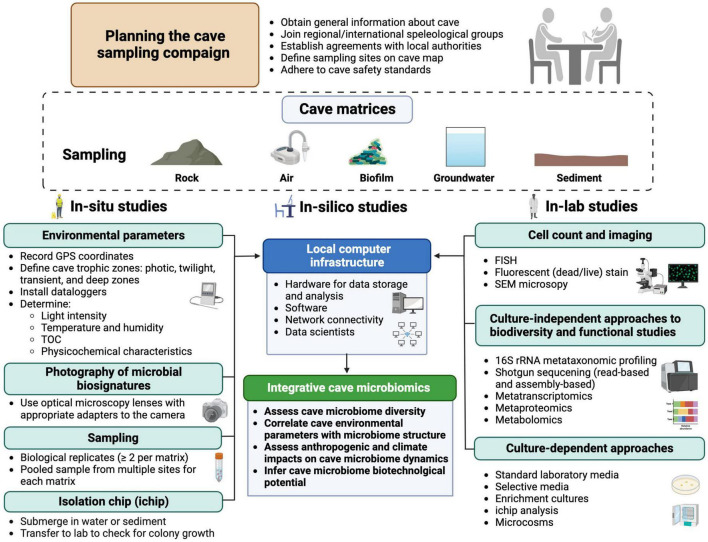
Integrated methodological workflow for cave microbiology research.

In the age of big data, integrative microbiome-based research is expected to provide a comprehensive understanding of microbiome changes and interactions by the assembly of datasets from the same ecological niche or sample type (Wood-Charlson et al., 2020). Different from human microbiome-based initiatives (e.g., The Integrative Hmp (iHMP) Research Network Consortium, 2019), no attempts have so far been made to apply integrative microbiomics to cave microbiology. Caves are diverse but relatively stable ecosystems, so they could be suited to integrative studies. Physicochemical characterization datasets and multi-omics studies from a variety of cave types could be integrated into a standardized database and made accessible to cave scientists (e.g., microbiologists, geologists, physicists, climatologists, etc.) (Figure 9). Various strategies can be used, including a number of statistical tests for microbiome differential abundance analyses (Lin and Peddada, 2020), spectral clustering (Imangaliyev et al., 2015), and network analyses (Jiang et al., 2019). Bioinformatic tools have also been proposed for the integrative analysis of microbiome datasets, which could well apply to cave microbiome research (The Integrative Hmp (iHMP) Research Network Consortium, 2019; Buza et al., 2019). This concept can also be useful for monitoring cave microbiome biodiversity over time and detecting the effects of climate change and the anthropogenic impact, in addition to predicting potential biotechnological applications of cave microorganisms. A connection between cave microbiomes and surface climatic conditions was inferred from the analysis of various terrestrial caves across the globe, highlighting the sensitivity of cave microbial communities to changes in external environmental conditions (Biagioli et al., 2024). In the future, combining environmental and microbial genomics data with machine learning algorithms will improve biomonitoring (Cordier et al., 2019), and provide new insights into the microbial ecology of cave systems. Noteworthy, machine learning tackled fundamental problems in the ecology of complex microbial communities (Wang et al., 2024), and machine-learning algorithms have already been used in the prediction of cave entrances on the Earth and on Mars (Character et al., 2023; Watson and Baldini, 2024).

## 7 Conclusion

Caves are increasingly attractive to microbiological investigation, as they represent geologically diverse environments unified by extreme conditions for life. Microbial communities composed of bacteria, archaea, fungi, and micro-eukaryotes are the living backbone of cave biota and play a key role in sustaining trophic networks. Studies of cave microbiomes have mostly been focused on the geomicrobiological interactions with different rock matrices, microbial diversity, mechanisms of microbial resistance and adaptation, anthropogenic impact on the cave ecosystem, and the biotechnological potential of cave microorganisms. Limestone, SAS, and volcanic caves have different genesis, textures of the rock matrix, and chemical composition, which determine different patterns of microbial colonization. Sulfidic caves provide an energetically exploitable ecosystem to chemolithoautotrophic metabolism due to the presence of H_2_S-rich groundwater, determining the predominance of sulfur-oxidizing *Campylobacterota*. Limestone caves are typically oligotrophic environments, while volcanic caves are shallow subsurface voids easily accessible by nutrients, and both cave types are primarily colonized by *Pseudomonadota* and *Actinomycetota*.

*Actinomycetota* are particularly abundant in volcanic caves and are endowed with great biotechnological potential. These bacteria could be isolated as pure cultures, and their ability to produce added-value secondary metabolites such as antibiotics and enzymes could be exploited under laboratory conditions. Yet, a significant portion of cave microbiomes is composed of still unclassified and uncharacterized taxa which deserve more focused investigation in search of new species and new metabolic functions or pathways.

Caves are difficult-to-access sites for untrained microbiologists, so it is not surprising that most studies have focused on microbial communities sampled near entrances or in caves with sub-horizontal development, in the so-called “transition zone”, where the environmental parameters vary depending on external climatic conditions. Conversely, the deepest cave zones are largely unexplored, although they account for a huge surface of underground voids. Stable environmental conditions and nutrient scarcity make deep zones an attractive model to study life under conditions of extreme oligotrophy and may also provide useful hints on how life functioned on the early Earth or how microorganisms might live beneath the surface of Mars or other planets.

The rock texture and chemical composition certainly contribute to inter-cave microbiome variability, being affected by the compactness and porosity properties of limestone and volcanic rocks, respectively. Attempts to define a core microbiome for geologically similar cave types have been successful for limestone caves (Biagioli et al., 2023), but not for volcanic caves (Gonzalez-Pimentel et al., 2018). However, genuinely autochthonous cave microorganisms can hardly be defined without robust comparative studies that differentiate them from allochthonous ones, i.e., those driven from outside through infiltrating water, bat guano, soil, air, etc. Meta-analysis of microbial community structures from different cave types could therefore help better define shared taxa between different types of caves, but this approach is challenging. For instance, Sequence Read Archive (SRA) data and accessory metadata (e.g., cave type, cave zones, environmental matrix, etc.) are unavailable in major sequence repositories (e.g., NCBI) for many studies. Moreover, eDNA extraction methods, 16S rRNA regions used as taxonomic markers, and sequencing technologies greatly vary among studies, complicating the assembly of a reliable dataset for comparative analysis. It should also be emphasized that the 16S rRNA-barcoding approach can only determine the taxonomic composition of the cave microbiome, but it does not provide information on its functional capabilities, with some arguing that a taxonomic approach is no longer useful and that a core functional microbiome should be prioritized (Neu et al., 2021).

Due to the high inter-cave variability and the complex dynamics of cave microbiomes, several key considerations are crucial for conducting statistically robust comparative studies at lower taxonomic levels, including sample size, replication, data compositionality, and cohorts’ population. Future comparative studies should also address the taxonomic and potentially functional characteristics of cave microbial communities by utilizing a statistically significant number of shotgun sequencing samples obtained from caves. Additional efforts should therefore be directed to the harmonization of cave microbiome studies, encompassing all steps of the investigation, from the sampling campaign to downstream data analyses.

## Author contributions

PT: Conceptualization, Data curation, Formal analysis, Investigation, Methodology, Validation, Visualization, Writing – original draft, Writing – review and editing. AC: Conceptualization, Data curation, Formal analysis, Investigation, Methodology, Validation, Visualization, Writing – original draft, Writing – review and editing. FPR: Data curation, Formal analysis, Investigation, Writing – review and editing. PV: Conceptualization, Formal analysis, Investigation, Funding acquisition, Methodology, Project administration, Resources, Supervision, Validation, Visualization, Writing – original draft, Writing – review and editing.

## References

[B1] Addesso R. Gonzalez-Pimentel J. L. D’Angeli I. M. De Waele J. Saiz-Jimenez C. Jurado V. (2021). Microbial community characterizing vermiculations from karst caves and its role in their formation. *Microb. Ecol.* 81 884–896. 10.1007/s00248-020-01623-5 33156395 PMC8062384

[B2] Alonso L. Pommier T. Kaufmann B. Dubost A. Chapulliot D. Doré J. (2019). Anthropization level of Lascaux Cave microbiome shown by regional-scale comparisons of pristine and anthropized caves. *Mol. Ecol.* 28 3383–3394. 10.1111/mec.15144 31177607

[B3] Anda D. Szabó A. Kovács-Bodor P. Makk J. Felföldi T. Ács É (2020). In situ modelling of biofilm formation in a hydrothermal spring cave. *Sci. Rep.* 10:21733. 10.1038/s41598-020-78759-4 33303927 PMC7729855

[B4] Asnicar F. Thomas A. M. Beghini F. Mengoni C. Manara S. Manghi P. (2020). Precise phylogenetic analysis of microbial isolates and genomes from metagenomes using PhyloPhlAn 3.0. *Nat. Commun.* 11:2500. 10.1038/s41467-020-16366-7 32427907 PMC7237447

[B5] Badino G. (2010). Underground meteorology– “What’s the weather underground?”. *Acta Carsol.* 39 427–448. 10.3986/ac.v39i3.74 17326625

[B6] Barka E. A. Vatsa P. Sanchez L. Gaveau-Vaillant N. Jacquard C. Meier-Kolthoff J. P. (2016). Taxonomy, physiology, and natural products of Actinobacteria. *Microbiol. Mol. Biol. Rev.* 80 1–43. 10.1128/mmbr.00019-15 26609051 PMC4711186

[B7] Barton H. (2015). “Starving artists: Bacterial oligotrophic heterotrophy in caves,” in *Microbial life of cave systems*, ed. Engel A. S. (Boston, MA: De Gruyter), 79–104.

[B8] Barton H. A. (2006). Introduction to cave microbiology: A review for the non-specialist. *J. Cave Karst Stud.* 68 43–54.

[B9] Barton H. A. Jurado V. (2007). What’s up down there? Microbial diversity in caves. *Microbe* 2 132–138.

[B10] Barton H. A. Luiszer F. (2005). Microbial metabolic structure in a sulfidic cave hot spring: Potential mechanisms of biospeleogenesis. *J. Cave Karst Stud.* 67 28–38.

[B11] Barton H. A. Northup D. E. (2007). Geomicrobiology in cave environments: Past, current and future perspectives. *J. Cave Karst Stud.* 69 163–178.

[B12] Bashiardes S. Zilberman-Schapira G. Elinav E. (2016). Use of metatranscriptomics in microbiome research. *Bioinform. Biol. Insights* 10 19–25. 10.4137/BBI.S34610 27127406 PMC4839964

[B13] Behrendt L. Trampe E. L. Nord N. B. Nguyen J. Kühl M. Lonco D. (2020). Life in the dark: Far-red absorbing cyanobacteria extend photic zones deep into terrestrial caves. *Environ. Microbiol.* 22 952–963. 10.1111/1462-2920.14774 31390129

[B14] Bender K. E. Glover K. Archey A. Barton H. A. (2020). The impact of sample processing and media chemistry on the culturable diversity of bacteria isolated from a cave. *Int. J. Speleol.* 49 209–220. 10.5038/1827-806X.49.3.2337

[B15] Bendia A. G. Callefo F. Araújo M. N. Sanchez E. Teixeira V. C. Vasconcelos A. (2022). Metagenome-assembled genomes from Monte Cristo cave (Diamantina, Brazil) reveal prokaryotic lineages as functional models for life on Mars. *Astrobiology* 22 293–312. 10.1089/ast.2021.0016 34694925

[B16] Beng K. C. Corlett R. T. (2020). Applications of environmental DNA (eDNA) in ecology and conservation: Opportunities, challenges and prospects. *Biodivers. Conserv.* 29 2089–2121. 10.1007/s10531-020-01980-0

[B17] Benoit J. B. Yoder J. A. Zettler L. W. Hobbs H. H. (2004). Mycoflora of a trogloxenic cave cricket, *Hadenoecus cumberlandicus* (Orthoptera: Rhaphidophoridae), from two small caves in northeastern Kentucky. *Ann. Entomol. Soc. Am.* 97 989–993. 10.1603/0013-87462004097[0989:MOATCC]2.0.CO;2

[B18] Bhullar K. Waglechner N. Pawlowski A. Koteva K. Banks E. D. Johnston M. D. (2012). Antibiotic resistance is prevalent in an isolated cave microbiome. *PLoS One* 7:e34953. 10.1371/journal.pone.0034953 22509370 PMC3324550

[B19] Biagioli F. Coleine C. Delgado-Baquerizo M. Feng Y. Saiz-Jimenez C. Selbmann L. (2024). Outdoor climate drives diversity patterns of dominant microbial taxa in caves worldwide. *Sci. Total Environ.* 906:167674. 10.1016/j.scitotenv.2023.167674 37813267

[B20] Biagioli F. Coleine C. Piano E. Nicolosi G. Poli A. Prigione V. (2023). Microbial diversity and proxy species for human impact in Italian karst caves. *Sci. Rep.* 13:689. 10.1038/s41598-022-26511-5 36639707 PMC9839721

[B21] Bin L. Ye C. Lijun Z. Ruidong Y. (2008). Effect of microbial weathering on carbonate rocks. *Earth Sci. Front.* 15 90–99. 10.1016/S1872-5791(09)60009-9

[B22] Bodor A. Bounedjoum N. Vincze G. E. Erdeiné Kis Á Laczi K. Szilágyi G. (2020). Challenges of unculturable bacteria: Environmental perspectives. *Rev. Environ. Sci. Biotechnol.* 9 1–22. 10.1007/s11157-020-09522-4

[B23] Bogdan D. F. Baricz A. I. Chiciudean I. Bulzu P. A. Cristea A. Năstase-Bucur R. (2023). Diversity, distribution and organic substrates preferences of microbial communities of a low anthropic activity cave in North-Western Romania. *Front. Microbiol.* 14:962452. 10.3389/fmicb.2023.962452 36825091 PMC9941645

[B24] Bontemps Z. Alonso L. Pommier T. Hugoni M. Moënne-Loccoz Y. (2022). Microbial ecology of tourist Paleolithic caves. *Sci. Total Environ.* 816:151492. 10.1016/j.scitotenv.2021.151492 34793801

[B25] Brad T. Iepure S. Sarbu S. M. (2021). The chemoautotrophically based Movile Cave groundwater ecosystem, a hotspot of subterranean biodiversity. *Diversity* 13:128. 10.3390/d13030128

[B26] Buza T. M. Tonui T. Stomeo F. Tiambo C. Katani R. Schilling M. (2019). iMAP: An integrated bioinformatics and visualization pipeline for microbiome data analysis. *BMC Bioinformatics* 20:374. 10.1186/s12859-019-2965-4 31269897 PMC6610863

[B27] Cacchio P. Ferrini G. Ercole C. Del Gallo M. Lepidi A. (2014). Biogenicity and characterization of moonmilk in the Grotta Nera (Majella National Park, Abruzzi, Central Italy). *J Cave Karst Stud.* 76 88–103. 10.4311/2012MB0275

[B28] Cañaveras J. C. Cuezva S. Sanchez-Moral S. Lario J. Laiz L. Gonzalez J. M. (2006). On the origin of fiber calcite crystals in moonmilk deposits. *Naturwissenschaften* 93 27–32. 10.1007/s00114-005-0052-3 16240102

[B29] Carmichael S. K. Zorn B. T. Santelli C. M. Roble L. A. Carmichael M. J. Bräuer S. L. (2015). Nutrient input influences fungal community composition and size and can stimulate manganese (II) oxidation in caves. *Environ. Microbiol. Rep.* 7 592–605. 10.1111/1758-2229.12291 25865809

[B30] Carvalho F. M. Souza R. C. Barcellos F. G. Hungria M. Vasconcelos A. T. R. (2010). Genomic and evolutionary comparisons of diazotrophic and pathogenic bacteria of the order Rhizobiales. *BMC Microbiol.* 10:37. 10.1186/1471-2180-10-37 20144182 PMC2907836

[B31] Caumartin V. (1963). Review of the microbiology of underground environments. *Natl. Speleol. Soc. Bull.* 25 1–14.

[B32] Character L. D. Beach T. Luzzadder-Beach S. Cook D. Schank C. Valdez F. Jr. (2023). Machine learning for cave entrance detection in a Maya archaeological area. *Phys. Geogr.* 10.1080/02723646.2023.2261182

[B33] Chen J. S. Tsai H. C. Hsu B. M. Fan C. W. Fang C. Y. Huang T. Y. (2021). The role of bacterial community in the formation of a stalactite in coral limestone areas of Taiwan by 16S rRNA gene amplicon surveys. *Environ. Earth Sci.* 80:665. 10.1007/s12665-021-09969-w

[B34] Cheng X. Xiang X. Yun Y. Wang W. Wang H. Bodelier P. L. (2023). Archaea and their interactions with bacteria in a karst ecosystem. *Front. Microbiol.* 14:1068595. 10.3389/fmicb.2023.1068595 36814573 PMC9939782

[B35] Chiciudean I. Russo G. Bogdan D. F. Levei E. A. Faur L. Hillebrand-Voiculescu A. (2022). Competition-cooperation in the chemoautotrophic ecosystem of Movile Cave: First metagenomic approach on sediments. *Environ. Microbiome* 17:44. 10.1186/s40793-022-00438-w 35978381 PMC9386943

[B36] Cordier T. Lanzén A. Apothéloz-Perret-Gentil L. Stoeck T. Pawlowski J. (2019). Embracing environmental genomics and machine learning for routine biomonitoring. *Trends Microbiol.* 27 387–397. 10.1016/j.tim.2018.10.012 30554770

[B37] Covington B. C. Spraggins J. M. Ynigez-Gutierrez A. E. Hylton Z. B. Bachmann B. O. (2018). Response of secondary metabolism of hypogean actinobacterial genera to chemical and biological stimuli. *Appl. Environ. Microbiol.* 84 e01125-18. 10.1128/AEM.01125-18 30030223 PMC6146984

[B38] Culver D. C. Pipan T. (2009). *The biology of caves and other subterranean habitats.* Oxford: Oxford University Press.

[B39] D’Angeli I. M. Ghezzi D. Leuko S. Firrincieli A. Parise M. Fiorucci A. (2019a). Geomicrobiology of a seawater-influenced active sulfuric acid cave. *PLoS One* 14:e0220706. 10.1371/journal.pone.0220706 31393920 PMC6687129

[B40] D’Angeli I. M. Parise M. Vattano M. Madonia G. Galdenzi S. De Waele J. (2019b). Sulfuric acid caves of Italy: A review. *Geomorphology* 333 105–122. 10.1016/j.geomorph.2019.02.025

[B41] D’Angeli I. M. Serrazanetti D. I. Montanari C. Vannini L. Gardini F. De Waele J. (2017). Geochemistry and microbial diversity of cave waters in the gypsum karst aquifers of Emilia-Romagna region, Italy. *Sci. Total Environ.* 598 538–552. 10.1016/j.scitotenv.2017.03.270 28448941

[B42] D’Auria G. Artacho A. Rojas R. A. Bautista J. S. Méndez R. Gamboa M. T. (2018). Metagenomics of bacterial diversity in villa Luz caves with sulfur water springs. *Genes* 9:55. 10.3390/genes9010055 29361802 PMC5793206

[B43] D’Angelo T. Goordial J. Lindsay M. R. McGonigle J. Booker A. Moser D. (2023). Replicated life-history patterns and subsurface origins of the bacterial sister phyla Nitrospirota and Nitrospinota. *ISME J.* 17 891–902. 10.1038/s41396-023-01397-x 37012337 PMC10203281

[B44] de Bruin S. Vasquez-Cardenas D. Sarbu S. M. Meysman F. J. R. Sousa D. Z. van Loosdrecht M. C. M. (2022). Sulfated glycosaminoglycan-like polymers are present in an acidophilic biofilm from a sulfidic cave. *Sci. Total Environ.* 829:154472. 10.1016/j.scitotenv.2022.154472 35276175

[B45] De Kumar A. Muthiyan R. Sunder J. Bhattacharya D. Kundu A. Roy S. (2019). Profiling bacterial diversity of B2 cave, a limestone cave of Baratang, Andaman and Nicobar Islands, India. *Proc. Indian Natl. Sci. Acad.* 85 853–862. 10.16943/ptinsa/2019/49589

[B46] De Waele J. Gutierrez F. (2022). *Karst hydrogeology, geomorphology and caves.* Chichester: Wiley Blackwell.

[B47] Derewacz D. K. McNees C. R. Scalmani G. Covington C. L. Shanmugam G. Marnett L. J. (2014). Structure and stereochemical determination of hypogeamicins from a cave-derived actinomycete. *J. Nat. Prod.* 77 1759–1763. 10.1021/np400742p 25046128 PMC4334282

[B48] Dhami N. K. Mukherjee A. Watkin E. L. (2018). Microbial diversity and mineralogical-mechanical properties of calcitic cave speleothems in natural and in vitro biomineralization conditions. *Front. Microbiol.* 9:40. 10.3389/fmicb.2018.00040 29472898 PMC5810276

[B49] Djebaili R. Mignini A. Vaccarelli I. Pellegrini M. Spera D. M. Del Gallo M. (2022). Polyhydroxybutyrate-producing cyanobacteria from lampenflora: The case study of the “Stiffe” caves in Italy. *Front. Microbiol.* 13:933398. 10.3389/fmicb.2022.933398 35966678 PMC9366245

[B50] Dong Y. Gao J. Wu Q. Ai Y. Huang Y. Wei W. (2020). Co-occurrence pattern and function prediction of bacterial community in karst cave. *BMC Microbiol.* 20:137. 10.1186/s12866-020-01806-7 32471344 PMC7257168

[B51] Duncan T. R. Werner-Washburne M. Northup D. E. (2021). Diversity of siderophore-producing bacterial cultures from Carlsbad Caverns national park caves, Carlsbad, New Mexico. *J. Cave Karst Stud.* 83 29–43. 10.4311/2019ES0118 34556971 PMC8455092

[B52] Egemeier S. J. (1981). Cavern development by thermal waters. *Natl. Speleol. Soc. Bull.* 43 31–51.

[B53] Engel A. S. (2007). Observations on the biodiversity of sulfidic karst habitats. *J. Cave Karst Stud.* 69 187–206.

[B54] Engel A. S. (2010). “Microbial diversity of cave ecosystems,” in *Geomicrobiology: Molecular and environmental perspective*, eds Barton L. Mandl M. Loy A. (Dordrecht: Springer), 219–238.

[B55] Engel A. S. Lee N. Porter M. L. Stern L. A. Bennett P. C. Wagner M. (2003). Filamentous “Epsilonproteobacteria” dominate microbial mats from sulfidic cave springs. *Appl. Environ. Microbiol.* 69 5503–5511. 10.1128/AEM.69.9.5503-5511.2003 12957939 PMC194925

[B56] Engel A. S. Stern L. A. Bennett P. C. (2004b). Microbial contributions to cave formation: New insights into sulfuric acid speleogenesis. *Geology* 32 369–372. 10.1130/G20288.1

[B57] Engel A. S. Porter M. L. Stern L. A. Quinlan S. Bennett P. C. (2004a). Bacterial diversity and ecosystem function of filamentous microbial mats from aphotic (cave) sulfidic springs dominated by chemolithoautotrophic “Epsilonproteobacteria”. *FEMS Microbiol. Ecol.* 51 31–53. 10.1016/j.femsec.2004.07.004 16329854

[B58] Enyedi N. T. Anda D. Borsodi A. K. Szabó A. Pál S. E. Óvári M. (2019). Radioactive environment adapted bacterial communities constituting the biofilms of hydrothermal spring caves (Budapest, Hungary). *J. Environ. Radioact.* 203 8–17. 10.1016/j.jenvrad.2019.02.010 30844681

[B59] Espino del Castillo A. Beraldi-Campesi H. Amador-Lemus P. Beltrán H. I. Le Borgne S. (2018). Bacterial diversity associated with mineral substrates and hot springs from caves and tunnels of the Naica underground system (Chihuahua, Mexico). *Int. J. Speleol.* 47 213–227. 10.5038/1827-806X.47.2.2161

[B60] Falasco E. Ector L. Isaia M. Wetzel C. E. Hoffmann L. Bona F. (2014). Diatom flora in subterranean ecosystems: A review. *Internat. J. Speleol.* 43 231–251. 10.5038/1827-806X.43.3.1

[B61] Farda B. Djebaili R. Vaccarelli I. Del Gallo M. Pellegrini M. (2022a). Actinomycetes from caves: An overview of their diversity, biotechnological properties, and insights for their use in soil environments. *Microorganisms* 10:453. 10.3390/microorganisms10020453 35208907 PMC8875103

[B62] Farda B. Vaccarelli I. Ercole C. Djebaili R. Del Gallo M. Pellegrini M. (2022b). Exploring structure, microbiota, and metagenome functions of epigean and hypogean black deposits by microscopic, molecular and bioinformatic approaches. *Sci. Rep.* 12:19405. 10.1038/s41598-022-24159-9 36371463 PMC9653421

[B63] Fierer N. Bradford M. A. Jackson R. B. (2007). Toward an ecological classification of soil bacteria. *Ecology* 88 1354–1364. 10.1890/05-1839 17601128

[B64] Ford D. C. Williams P. (2007). *Karst hydrogeology and geomorphology.* Chichester: Wiley.

[B65] Forti P. Galdenzi S. Sarbu S. M. (2002). The hypogenic caves: A powerful tool for the study of seeps and their environmental effects. *Cont. Shelf Res.* 22 2373–2386. 10.1016/S0278-4343(02)00062-6

[B66] Ghezzi D. Foschi L. Firrincieli A. Hong P. Y. Vergara F. De Waele J. (2022). Insights into the microbial life in silica-rich subterranean environments: Microbial communities and ecological interactions in an orthoquartzite cave (Imawarì Yeuta, Auyan Tepui, Venezuela). *Front. Microbiol.* 13:930302. 10.3389/fmicb.2022.930302 36212823 PMC9537377

[B67] Ghosh S. Kuisiene N. Cheeptham N. (2017a). The cave microbiome as a source for drug discovery: Reality or pipe dream? *Biochem. Pharmacol.* 134 18–34. 10.1016/j.bcp.2016.11.018 27867014

[B68] Ghosh S. Paine E. Wall R. Kam G. Lauriente T. Sa-Ngarmangkang P. C. (2017b). In situ cultured bacterial diversity from iron curtain cave, Chilliwack, British Columbia, Canada. *Diversity* 9:36. 10.3390/d9030036

[B69] Gillieson D. S. (2021). *Caves: Processes, development, and management.* Hoboken, NJ: Wiley-Blackwell.

[B70] Glime J. M. (2022). *“Caves,” bryophyte ecology: Habitat and role.* Houghton, MI: Michigan Technological University.

[B71] Gnaspini P. Trajano E. (2000). “Guano communities in tropical caves,” in *Ecosystems of the world*, eds Wilkens H. Culver D. C. Humphreys W. F. (Amsterdam: Elsevier), 251–268.

[B72] Goldscheider N. Chen Z. Auler A. S. Bakalowicz M. Broda S. Drew D. (2020). Global distribution of carbonate rocks and karst water resources. *Hydrogeol. J.* 28 1661–1677. 10.1007/s10040-020-02139-5

[B73] Gonzalez-Pimentel J. L. Hermosin B. Saiz-Jimenez C. Jurado V. (2022). *Streptomyces benahoarensis* sp. nov. isolated from a lava tube of la Palma, Canary Islands, Spain. *Front. Microbiol.* 13:907816. 10.3389/fmicb.2022.907816 35651486 PMC9149447

[B74] Gonzalez-Pimentel J. L. Martin-Pozas T. Jurado V. Miller A. Z. Caldeira A. T. Fernandez-Lorenzo O. (2021). Prokaryotic communities from a lava tube cave in La Palma Island (Spain) are involved in the biogeochemical cycle of major elements. *Peer J.* 9:e11386. 10.7717/peerj.11386 34026356 PMC8121065

[B75] Gonzalez-Pimentel J. L. Miller A. Z. Jurado V. Laiz L. Pereira M. F. Saiz-Jimenez C. (2018). Yellow coloured mats from lava tubes of La Palma (Canary Islands, Spain) are dominated by metabolically active Actinobacteria. *Sci. Rep.* 8:1944. 10.1038/s41598-018-20393-2 29386569 PMC5792456

[B76] Gosse J. T. Ghosh S. Sproule A. Overy D. Cheeptham N. Boddy C. N. (2019). Whole genome sequencing and metabolomic study of cave streptomyces isolates ICC1 and ICC4. *Front. Microbiol.* 10:1020. 10.3389/fmicb.2019.01020 31134037 PMC6524458

[B77] Griebler C. Malard F. Lefébure T. (2014). Current developments in groundwater ecology from biodiversity to ecosystem function and services. *Curr. Opin. Biotechnol.* 27 159–167. 10.1016/j.copbio.2014.01.018 24590188

[B78] Groth I. Vettermann R. Schuetze B. Schumann P. Sáiz-Jiménez C. (1999). Actinomycetes in karstic caves of northern Spain (Altamira and Tito Bustillo). *J. Microbiol. Methods* 36 115–122. 10.1016/S0167-7012(99)00016-0 10353805

[B79] Gulden B. (2019). *USA long cave list*. Available online at: https://cave-exploring.com/index.php/long-and-deep-caves-of-the-world/usa-long-cave-list/ (accessed December 15, 2023).

[B80] Gulecal-Pektas Y. Temel M. (2017). A Window to the subsurface: Microbial diversity in hot springs of a sulfidic cave (Kaklik, Turkey). *Geomicrobiol. J.* 34 374–384. 10.1080/01490451.2016.1204374

[B81] Hamedi J. Kafshnouchi M. Ranjbaran M. (2019). A study on actinobacterial diversity of Hampoeil cave and screening of their biological activities. *Saudi J. Biol. Sci.* 26 1587–1595. 10.1016/j.sjbs.2018.10.010 31762631 PMC6864206

[B82] Hathaway J. J. M. Garcia M. G. Balasch M. M. Spilde M. N. Stone F. D. Dapkevicius M. D. L. N. (2014). Comparison of bacterial diversity in Azorean and Hawai’ian lava cave microbial mats. *Geomicrobiol. J.* 31 205–220. 10.1080/01490451.2013.777491 26924866 PMC4765387

[B83] He D. Wu F. Ma W. Zhang Y. Gu J. D. Duan Y. (2021). Insights into the bacterial and fungal communities and microbiome that causes a microbe outbreak on ancient wall paintings in the Maijishan Grottoes. *Int. Biodeter. Biodegr.* 163:105250. 10.1016/j.ibiod.2021.105250

[B84] Herold K. Gollmick F. A. Growth I. Roth M. Menzel K. D. Mollmann U. (2005). Cervimycin A-D: A polyketide glycoside complex from a cave bacterium can defeat vancomycin resistance. *Chem. Eur. J.* 11 5523–5530. 10.1002/chem.200500320 15940739

[B85] Hershey O. S. Barton H. A. (2018). “The microbial diversity of caves,” in *Ecological studies*, eds Moldovan O. Kováč L. Halse S. (Cham: Springer), 69–90.

[B86] Hershey O. S. Kallmeyer J. Barton H. A. (2019). “A practical guide to studying the microbiology of karst aquifers,” in *Karst water environment: Advances in research, management and policy*, eds Younos T. Schreiber M. Kosič Ficco K. (Cham: Springer), 191–207.

[B87] Hershey O. S. Kallmeyer J. Wallace A. Barton M. D. Barton H. A. (2018). High microbial diversity despite extremely low biomass in a deep karst aquifer. *Front. Microbiol.* 9:2823. 10.3389/fmicb.2018.02823 30534116 PMC6275181

[B88] Hose L. D. Palmer A. N. Palmer M. V. Northup D. E. Boston P. J. DuChene H. R. (2000). Microbiology and geochemistry in a hydrogen-sulphide-rich karst environment. *Chem. Geol.* 169 399–423. 10.1016/S0009-2541(00)00217-5

[B89] Howarth F. G. (1980). The zoogeography of specialized cave animals: A bioclimatic model. *Evolution* 34 394–406. 10.2307/2407402 28563430

[B90] Howarth F. G. (2021). Glacier caves: A globally threatened subterranean biome. *J. Cave Karst Stud.* 83 66–70. 10.4311/2019LSC0132

[B91] Hutchins B. T. Engel A. S. Nowlin W. H. Schwartz B. F. (2016). Chemolithoautotrophy supports macroinvertebrate food webs and affects diversity and stability in groundwater communities. *Ecology* 97 1530–1542. 10.1890/15-1129.1 27459783

[B92] Idle J. R. Gonzalez F. J. (2007). Metabolomics. *Cell Metab.* 6 348–351. 10.1016/j.cmet.2007.10.005 17983580 PMC2140247

[B93] Imangaliyev S. Keijser B. Crielaard W. Tsivtsivadze E. (2015). Personalized microbial network inference via co-regularized spectral clustering. *Methods* 83 28–35. 10.1016/j.ymeth.2015.03.017 25842007

[B94] Jiang D. Armour C. R. Hu C. Mei M. Tian C. Sharpton T. J. (2019). Microbiome multi-omics network analysis: Statistical considerations, limitations, and opportunities. *Front. Genet.* 10:995. 10.3389/fgene.2019.00995 31781153 PMC6857202

[B95] Jiang L. Pu H. Xiang J. Su M. Yan X. Yang D. (2018). Huanglongmycin A-C, cytotoxic polyketides biosynthesized by a putative type II polyketide synthase from *Streptomyces* sp. *Front. Chem.* 6:254. 10.3389/fchem.2018.00254 30013965 PMC6036704

[B96] Jones A. A. Bennett P. C. (2014). Mineral microniches control the diversity of subsurface microbial populations. *Geomicrobiol. J.* 31 246–261. 10.1080/01490451.2013.809174

[B97] Jones D. S. Albrecht H. L. Dawson K. S. Schaperdoth I. Freeman K. H. Pi Y. (2012). Community genomic analysis of an extremely acidophilic sulfur-oxidizing biofilm. *ISME J.* 6 158–170. 10.1038/ismej.2011.75 21716305 PMC3246232

[B98] Jones D. S. Northup D. E. (2021). Cave decorating with microbes: Geomicrobiology of caves. *Elements* 17 107–112. 10.2138/gselements.17.2.107

[B99] Jones D. S. Lyon E. H. Macalady J. L. (2008). Geomicrobiology of biovermiculations from the Frasassi cave system. *Italy. J. Cave Karst Stud.* 70 78–93.

[B100] Jones D. S. Schaperdoth I. Macalady J. L. (2016). Biogeography of sulfur-oxidizing Acidithiobacillus populations in extremely acidic cave biofilms. *ISME J.* 10 2879–2891. 10.1038/ismej.2016.74 27187796 PMC5148195

[B101] Jones D. S. Schaperdoth I. Northup D. E. Gómez-Cruz R. Macalady J. L. (2023). Convergent community assembly among globally separated acidic cave biofilms. *Appl. Environ. Microbiol.* 89:e0157522. 10.1128/aem.01575-22 36602326 PMC9888236

[B102] Jones D. S. Tobler D. J. Schaperdoth I. Mainiero M. Macalady J. L. (2010). Community structure of subsurface biofilms in the thermal sulfidic caves of Acquasanta Terme, Italy. *Appl. Environ. Microbiol.* 76 5902–5910. 10.1128/AEM.00647-10 20639361 PMC2935061

[B103] Jurado V. D’Angeli I. Martin-Pozas T. Cappelletti M. Ghezzi D. Gonzalez-Pimentel J. L. (2021). Dominance of *Arcobacter* in the white filaments from the thermal sulfidic spring of Fetida Cave (Apulia, southern Italy). *Sci. Total Environ.* 800:149465. 10.1016/j.scitotenv.2021.149465 34391144

[B104] Jurado V. Gonzalez-Pimentel J. L. Miller A. Z. Hermosin B. D’Angeli I. M. Tognini P. (2020). Microbial communities in vermiculation deposits from an Alpine cave. *Front. Earth Sci.* 8:586248. 10.3389/feart.2020.586248

[B105] Kalam S. Basu A. Ahmad I. Sayyed R. Z. El-Enshasy H. A. Dailin D. J. (2020). Recent understanding of soil Acidobacteria and their ecological significance: A critical review. *Front. Microbiol.* 11:580024. 10.3389/fmicb.2020.580024 33193209 PMC7661733

[B106] Kashaf S. S. Almeida A. Segre J. A. Finn R. D. (2021). Recovering prokaryotic genomes from host-associated, short-read shotgun metagenomic sequencing data. *Nat. Protoc.* 16 2520–2541. 10.1038/s41596-021-00508-2 33864056

[B107] Keeler R. Lusk B. (2021). Microbiome of Grand Canyon caverns, a dry sulfuric karst cave in Arizona, supports diverse extremophilic bacterial and archaeal communities. *J. Cave Karst Stud.* 83 44–56. 10.4311/2019MB0126

[B108] Kempe S. (2019). “Volcanic rock caves,” in *Encyclopedia of caves*, 3rd Edn, eds White W. B. Culver D. C. Pipan T. (Amsterdam: Academic Press), 118–1127.

[B109] Kieraite-Aleksandrova I. Aleksandrovas V. Kuisiene N. (2015). Down into the earth: Microbial diversity of the deepest cave of the world. *Biologia* 70 989–1002. 10.1515/biolog-2015-0127

[B110] Kimble J. C. Winter A. S. Spilde M. N. Sinsabaugh R. L. Northup D. E. (2018). A potential central role of *Thaumarchaeota* in N-cycling in a semi-arid environment, fort Stanton cave, snowy river passage, New Mexico, USA. *FEMS Microbiol. Ecol.* 94:173. 10.1093/femsec/fiy173 30165514 PMC6669814

[B111] Klimchouk A. (2017). “Types and settings of Hypogene karst,” in *Hypogene karst regions and caves of the world*, Vol. 1 eds Klimchouk A. Palmer A. N. Waele J. D. Auler A. S. Audra P. (Cham: Springer), 1–39.

[B112] Klusaitė A. Vičkačkaitė V. Vaitkevičienė B. Karnickaitė R. Bukelskis D. Kieraitė-Aleksandrova I. (2016). Characterization of antimicrobial activity of culturable bacteria isolated from Krubera-Voronja Cave. *Int. J. Speleol.* 45 275–287.

[B113] Koner S. Chen J. S. Hsu B. M. Tan C. W. Fan C. W. Chen T. H. (2021). Assessment of carbon substrate catabolism pattern and functional metabolic pathway for microbiota of limestone caves. *Microorganisms* 9:1789. 10.3390/microorganisms9081789 34442868 PMC8398112

[B114] Kosznik-Kwaśnicka K. Golec P. Jaroszewicz W. Lubomska D. Piechowicz L. (2022). Into the unknown: Microbial communities in caves, their role, and potential use. *Microorganisms* 10:222. 10.3390/microorganisms10020222 35208677 PMC8877592

[B115] Kovaka S. Ou S. Jenike K. M. Schatz M. C. (2023). Approaching complete genomes, transcriptomes and epi-omes with accurate long-read sequencing. *Nat. Methods* 20 12–16. 10.1038/s41592-022-01716-8 36635537 PMC10068675

[B116] Kumaresan D. Wischer D. Stephenson J. Hillebrand-Voiculescu A. Murrell J. C. (2014). Microbiology of movile cave-a chemolithoautotrophic ecosystem. *Geomicrobiol. J.* 31 186–193. 10.1080/01490451.2013.839764

[B117] Latorre-Pérez A. Pascual J. Porcar M. Vilanova C. (2020). A lab in the field: Applications of real-time, in situ metagenomic sequencing. *Biol. Methods Protoc.* 5:baa016. 10.1093/biomethods/bpaa016 33134552 PMC7585387

[B118] Laurent D. Barré G. Durlet C. Cartigny P. Carpentier C. Paris G. (2023). Unravelling biotic versus abiotic processes in the development of large sulfuric-acid karsts. *Geology* 51 262–267. 10.1130/G50658.1

[B119] Lavoie K. H. Winter A. S. Read K. J. H. Hughes E. M. Spilde M. N. Northup D. E. (2017). Comparison of bacterial communities from lava cave microbial mats to overlying surface soils from lava beds national monument, USA. *PLoS One* 12:e0169339. 10.1371/journal.pone.0169339 28199330 PMC5310854

[B120] Lee J. Kim H. S. Jo H. Y. Kwon M. J. (2021). Revisiting soil bacterial counting methods: Optimal soil storage and pretreatment methods and comparison of culture-dependent and-independent methods. *PLoS One* 16:e0246142. 10.1371/journal.pone.0246142 33566842 PMC7875414

[B121] Lee N. M. Meisinger D. B. Aubrecht R. Kovacik L. Saiz-Jimenez C. Baskar S. (2012). “Caves and karst environments,” in *Life at extremes: Environments, organisms and strategies for survival*, ed. Bell E. M. (Wallingford: CAB International), 320–344.

[B122] Leuko S. Koskinen K. Sanna L. D’Angeli I. M. De Waele J. Marcia P. (2017). The influence of human exploration on the microbial community structure and ammonia-oxidizing potential of the Su Bentu limestone cave in Sardinia, Italy. *PLoS One* 12:e0180700. 10.1371/journal.pone.0180700 28704427 PMC5507542

[B123] Lin H. Peddada S. D. (2020). Analysis of microbial compositions: A review of normalization and differential abundance analysis. *NPJ Biofilms Microbiomes* 6:60. 10.1038/s41522-020-00160-w 33268781 PMC7710733

[B124] Liu Q. He Z. Naganuma T. Nakai R. Rodríguez L. M. Carreño R. (2022). Phylotypic diversity of bacteria associated with speleothems of a silicate cave in a Guiana Shield tepui. *Microorganisms* 10:1395. 10.3390/microorganisms10071395 35889113 PMC9316562

[B125] Long Y. Jiang J. Hu X. Zhou J. Hu J. Zhou S. (2019). Actinobacterial community in Shuanghe cave using culture-dependent and-independent approaches. *World J. Microbiol. Biotechnol.* 35:153. 10.1007/s11274-019-2713-y 31576426

[B126] Lücker S. Wagner M. Maixner F. Pelletier E. Koch H. Vacherie B. (2010). A Nitrospira metagenome illuminates the physiology and evolution of globally important nitrite-oxidizing bacteria. *Proc. Natl. Acad. Sci. U.S.A.* 107 13479–13484. 10.1073/pnas.1003860107 20624973 PMC2922143

[B127] Ma L. Huang X. Wang H. Yun Y. Cheng X. Liu D. (2021). Microbial interactions drive distinct taxonomic and potential metabolic responses to habitats in karst cave ecosystem. *Microbiol. Spectr.* 9:e0115221. 10.1128/Spectrum.01152-21 34494852 PMC8557908

[B128] Macalady J. L. Dattagupta S. Schaperdoth I. Jones D. S. Druschel G. K. Eastman D. (2008). Niche differentiation among sulfur-oxidizing bacterial populations in cave waters. *ISME J.* 2 590–601. 10.1038/ismej.2008.25 18356823

[B129] Macalady J. L. Jones D. S. Lyon E. H. (2007). Extremely acidic, pendulous cave wall biofilms from the Frasassi cave system, Italy. *Environ. Microbiol.* 9 1402–1414. 10.1111/j.1462-2920.2007.01256.x 17504478

[B130] Macalady J. L. Lyon E. H. Koffman B. Albertson L. K. Meyer K. Galdenzi S. (2006). Dominant microbial populations in limestone-corroding stream biofilms, Frasassi cave system, Italy. *Appl. Environ. Microbiol.* 72 5596–5609. 10.1128/AEM.00715-06 16885314 PMC1538711

[B131] Maciejewska M. Adam D. Martinet L. Naômé A. Całusińska M. Delfosse P. (2016). A phenotypic and genotypic analysis of the antimicrobial potential of cultivable Streptomyces isolated from cave moonmilk deposits. *Front. Microbiol.* 7:1455. 10.3389/fmicb.2016.01455 27708627 PMC5030222

[B132] Maciejewska M. Adam D. Naômé A. Martinet L. Tenconi E. Całusińska M. (2017). Assessment of the potential role of streptomyces in cave moonmilk formation. *Front. Microbiol.* 8:1181. 10.3389/fmicb.2017.01181 28706508 PMC5489568

[B133] Măntoiu D. Ş Mirea I. C. Şandric I. C. Cîşlariu A. G. Gherghel I. Constantin S. (2022). Bat dynamics modelling as a tool for conservation management in subterranean environments. *PLoS One* 17:e0275984. 10.1371/journal.pone.0275984 36264951 PMC9584375

[B134] Marques E. L. S. Silva G. S. Dias J. C. T. Gross E. Costa M. S. Rezende R. P. (2019). Cave drip water related samples as a natural environment for aromatic hydrocarbon-degrading bacteria. *Microorganisms* 7:33. 10.3390/microorganisms7020033 30691082 PMC6406655

[B135] Martin-Pozas T. Fernandez-Cortes A. Cuezva S. Cañaveras J. C. Benavente D. Duarte E. (2023). New insights into the structure, microbial diversity and ecology of yellow biofilms in a Paleolithic rock art cave (Pindal Cave, Asturias, Spain). *Sci. Total. Environ.* 897:165218. 10.1016/j.scitotenv.2023.165218 37419360

[B136] Martin-Pozas T. Nováková A. Jurado V. Fernandez-Cortes A. Cuezva S. Saiz-Jimenez C. (2022). Diversity of Microfungi in a high radon cave ecosystem. *Front. Microbiol.* 13:869661. 10.3389/fmicb.2022.869661 35572646 PMC9093739

[B137] McCluskey K. Barker K. B. Barton H. A. Boundy-Mills K. Brown D. R. Coddington J. A. (2017). The US culture collection network responding to the requirements of the Nagoya protocol on access and benefit sharing. *mBio* 8 e00982-17. 10.1128/mBio.00982-17 28811341 PMC5559631

[B138] Mejía-Ortíz L. Christman M. C. Pipan T. Culver D. C. (2020). What’s the temperature in tropical caves? *PLoS One.* 15:e0237051. 10.1371/journal.pone.0237051 33382693 PMC7775087

[B139] Michail G. Karapetsi L. Madesis P. Reizopoulou A. Vagelas I. (2021). Metataxonomic analysis of bacteria entrapped in a stalactite’s core and their possible environmental origins. *Microorganisms* 9:2411. 10.3390/microorganisms9122411 34946013 PMC8705861

[B140] Mondini A. Anwar M. Z. Ellegaard-Jensen L. Lavin P. Jacobsen C. S. Purcarea C. (2022). Heat shock response of the active microbiome from perennial cave ice. *Front. Microbiol.* 12:4370. 10.3389/fmicb.2021.809076 35360653 PMC8960993

[B141] Morgan I. M. (1991). *Geology of caves.* Washington, DC: U.S. Government Printing Office, 10.3133/7000072

[B142] Mueller A. J. Daebeler A. Herbold C. W. Kirkegaard R. H. Daims H. (2023). Cultivation and genomic characterization of novel and ubiquitous marine nitrite-oxidizing bacteria from the Nitrospirales. *ISME J.* 17 2123–2133. 10.1038/s41396-023-01518-6 37749300 PMC10579370

[B143] Mulec J. Oarga-Mulec A. Tomazin R. Matos T. (2015). Characterization and fluorescence of yellow biofilms in karst caves, southwest Slovenia. *Int. J. Speleol.* 44 107–114.

[B144] Neu A. T. Allen E. E. Roy K. (2021). Defining and quantifying the core microbiome: Challenges and prospects. *Proc. Natl. Acad. Sci. U.S.A.* 118:e2104429118. 10.1073/pnas.2104429118 34862327 PMC8713806

[B145] Nichols D. Cahoon N. Trakhtenberg E. M. Pham L. Mehta A. Belanger A. (2010). Use of ichip for high throughput in situ cultivation of “uncultivable” microbial species. *Appl. Environ. Microbiol.* 76 2445–2450. 10.1128/AEM.01754-09 20173072 PMC2849220

[B146] Nicolosi G. Gonzalez-Pimentel J. L. Piano E. Isaia M. Miller A. Z. (2023). First Insights into the bacterial diversity of Mount Etna volcanic caves. *Microb. Ecol.* 86 1632–1645. 10.1007/s00248-023-02181-2 36750476 PMC10497698

[B147] Northup D. E. Lavoie K. H. (2001). Geomicrobiology of caves: A review. *Geomicrobiol. J.* 18 199–222. 10.1080/01490450152467750

[B148] Northup D. E. Melim L. A. Spilde M. N. Hathaway J. J. Garcia M. G. Moya M. (2011). Lava cave microbial communities within mats and secondary mineral deposits: Implications for life detection on other planets. *Astrobiology* 11 601–618. 10.1089/ast.2010.0562 21879833 PMC3176350

[B149] Northup D. Lavoie K. (2015). “Microbial Diversity and ecology of lava caves,” in *Microbial life of cave systems*, ed. Engel A. S. (Boston, MA: De Gruyter), 161–192.

[B150] Novak T. Perc M. Lipovšek S. Janžekovič F. (2012). Duality of terrestrial subterranean fauna. *Int. J. Speleol.* 41 181–188. 10.5038/1827-806X.41.2.5

[B151] Nygaard A. B. Tunsjø H. S. Meisal R. Charnock C. (2020). A preliminary study on the potential of Nanopore MinION and illumina MiSeq 16S rRNA gene sequencing to characterize building-dust microbiomes. *Sci. Rep.* 10:3209. 10.1038/s41598-020-59771-0 32081924 PMC7035348

[B152] Oliveira C. Gunderman L. Coles C. A. Lochmann J. Parks M. Ballard E. (2017). 16S rRNA gene-based metagenomic analysis of Ozark cave bacteria. *Diversity* 9:31. 10.3390/d9030031 29551950 PMC5856467

[B153] Oren A. Garrity G. M. (2021). Valid publication of the names of forty-two phyla of prokaryotes. *Int. J. Syst. Evol. Microbiol.* 71:005056. 10.1099/ijsem.0.005056 34694987

[B154] Ortiz M. Legatzki A. Neilson J. W. Fryslie B. Nelson W. M. Wing R. A. (2014). Making a living while starving in the dark: Metagenomic insights into the energy dynamics of a carbonate cave. *ISME J.* 8 478–491. 10.1038/ismej.2013.159 24030597 PMC3906820

[B155] Palmer A. N. (1991). Origin and morphology of limestone caves. *Geol. Soc. Am. Bull.* 103 1–21. 10.1130/0016-76061991103<0001:OAMOLC<2.3.CO;2

[B156] Palmer A. N. (2007). *Cave geology.* Dayton, OH: Cave Books.

[B157] Palmer A. N. Hill C. A. (2019). “Sulfuric acid caves,” in *Encyclopedia of caves*, eds White W. B. Culver D. C. Pipan T. (Cambridge, MA: Academic Press), 1053–1062.

[B158] Parise M. Galeazzi C. Bixio R. Dixon M. (2013). “Classification of artificial cavities: A first contribution by the UIS Commission,” in *Proceedings of the 16th international congress of speleology BRNO*, (Prague: Czech Speleological Society), 230–235.

[B159] Park S. Cho Y. J. Jung D. Y. Jo K. N. Lee E. J. Lee J. S. (2020). Microbial diversity in moonmilk of Baeg-nyong cave, Korean CZO. *Front. Microbiol.* 11:613. 10.3389/fmicb.2020.00613 32390967 PMC7190796

[B160] Pašić L. Kovče B. Sket B. Herzog-Velikonja B. (2009). Diversity of microbial communities colonizing the walls of a Karstic cave in Slovenia. *FEMS Microbiol. Ecol.* 71 50–60. 10.1111/j.1574-6941.2009.00789.x 19817862

[B161] Pawlowski A. C. Wang W. Koteva K. Barton H. A. McArthur A. G. Wright G. D. (2016). A diverse intrinsic antibiotic resistome from a cave bacterium. *Nat. Commun.* 7:13803. 10.1038/ncomms13803 27929110 PMC5155152

[B162] Porca E. Jurado V. Martin-Sanchez P. M. Hermosin B. Bastian F. Alabouvette C. (2011). Aerobiology: An ecological indicator for early detection and control of fungal outbreaks in caves. *Ecol. Indic.* 11 1594–1598. 10.1016/j.ecolind.2011.04.003

[B163] Porca E. Jurado V. Žgur-Bertok D. Saiz-Jimenez C. Pašić L. (2012). Comparative analysis of yellow microbial communities growing on the walls of geographically distinct caves indicates a common core of microorganisms involved in their formation. *FEMS Microbiol. Ecol.* 81 255–266. 10.1111/j.1574-6941.2012.01383.x 22486654

[B164] Prescott R. D. Zamkovaya T. Donachie S. P. Northup D. E. Medley J. J. Monsalve N. (2022). Islands within islands: Bacterial phylogenetic structure and consortia in Hawaiian lava caves and fumaroles. *Front. Microbiol.* 13:934708. 10.3389/fmicb.2022.934708 35935195 PMC9349362

[B165] Pronk M. Goldscheider N. Zopfi J. Zwahlen F. (2009). Percolation and particle transport in the unsaturated zone of a karst aquifer. *Groundwater* 47 361–369. 10.1111/j.1745-6584.2008.00509.x 19462487

[B166] Qian Z. Mao Y. Xiong S. Peng B. Liu W. Liu H. (2020). Historical residues of organochlorine pesticides (OCPs) and polycyclic aromatic hydrocarbons (PAHs) in a flood sediment profile from the Longwang Cave in Yichang, China. *Ecotoxicol. Environ. Saf.* 196:110542. 10.1016/j.ecoenv.2020.110542 32276160

[B167] Quince C. Walker A. W. Simpson J. T. Loman N. J. Segata N. (2017). Shotgun metagenomics, from sampling to analysis. *Nat. Biotechnol.* 35 833–844. 10.1038/nbt.3935 28898207

[B168] Rachid A. N. Güngör N. D. (2022). Screening of bioactive compounds for biomedical and industrial uses from Actinobacteria isolated from the Parsik Cave (Turkey). *Johnson Matthey Technol. Rev.* 67 159–170. 10.1595/205651322X16482034395036

[B169] Rangseekaew P. Pathom-aree W. (2019). Cave Actinobacteria as producers of bioactive metabolites. *Front. Microbiol.* 10:387. 10.3389/fmicb.2019.00387 30967844 PMC6438885

[B170] Raynaud X. Nunan N. (2014). Spatial ecology of bacteria at the microscale in soil. *PLoS One* 9:e87217. 10.1371/journal.pone.0087217 24489873 PMC3905020

[B171] Reboleira A. S. Bodawatta K. H. Ravn N. M. R. Lauritzen S. E. Skoglund R. Ø Poulsen M. (2022). Nutrient-limited subarctic caves harbour more diverse and complex bacterial communities than their surface soil. *Environ. Microbiome* 17:41. 10.1186/s40793-022-00435-z 35941623 PMC9361705

[B172] Riquelme C. Dapkevicius M. Miller A. Charlop-Powers Z. Brady S. Mason C. (2017). Biotechnological potential of Actinobacteria from Canadian and Azorean volcanic caves. *Appl. Microbiol. Biotechnol.* 101 843–857. 10.1007/s00253-016-7932-7 27812802

[B173] Riquelme C. Marshall Hathaway J. J. Enes Dapkevicius M. L. Miller A. Z. Kooser A. Northup D. E. (2015). Actinobacterial diversity in volcanic caves and associated geomicrobiological interactions. *Front. Microbiol.* 6:1342. 10.3389/fmicb.2015.01342 26696966 PMC4673402

[B174] Rohwerder T. Sand W. Lascu C. (2003). Preliminary evidence for a sulphur cycle in Movile Cave, Romania. *Acta Biotechnol.* 23 101–107. 10.1002/abio.200390000

[B175] Roldán C. Murcia-Mascarós S. López-Montalvo E. Vilanova C. Porcar M. (2018). Proteomic and metagenomic insights into prehistoric Spanish Levantine rock art. *Sci Rep.* 8:10011. 10.1038/s41598-018-28121-6 29968740 PMC6030215

[B176] Rossmassler K. Engel A. S. Twing K. I. Hanson T. E. Campbell B. J. (2012). Drivers of epsilonproteobacterial community composition in sulfidic caves and springs. *FEMS Microbiol. Ecol.* 79 421–432. 10.1111/j.1574-6941.2011.01231.x 22092920

[B177] Rossmassler K. Hanson T. E. Campbell B. J. (2016). Diverse sulfur metabolisms from two subterranean sulfidic spring systems. *FEMS Microbiol. Lett.* 363 fnw162. 10.1093/femsle/fnw162 27324397

[B178] Sauro F. Cappelletti M. Ghezzi D. Columbu A. Hong P. Y. Zowawi H. M. (2018). Microbial diversity and biosignatures of amorphous silica deposits in orthoquartzite caves. *Sci. Rep.* 8:17569. 10.1038/s41598-018-35532-y 30514906 PMC6279750

[B179] Scharping R. J. Garey J. R. (2021). Relationship between aquifer biofilms and unattached microbial indicators of urban groundwater contamination. *Mol. Ecol.* 30 324–342. 10.1111/mec.15713 33113280

[B180] Simon K. S. (2019). “Cave ecosystems,” in *Encyclopedia of caves*, 3rd Edn, eds White W. B. Culver D. C. Pipan T. (Cambridge, MA: Academic Press), 223–226.

[B181] Simon K. S. Pipan T. Culver D. C. (2007). A conceptual model of the flow and distribution of organic carbon in caves. *J. Cave Karst Stud.* 69 279–284.

[B182] Snider J. R. Goin C. Miller R. V. Boston P. J. Northup D. E. (2009). Ultraviolet radiation sensitivity in cave bacteria: Evidence of adaptation to the subsurface? *Int. J. Speleol.* 38 11–22. 10.5038/1827-806X.38.1.2

[B183] Sogin E. M. Kleiner M. Borowski C. Gruber-Vodicka H. R. Dubilier N. (2021). Life in the dark: Phylogenetic and physiological diversity of chemosynthetic symbioses. *Annu. Rev. Microbiol.* 75 695–718. 10.1146/annurev-micro-051021-123130 34351792

[B184] Taylor M. L. Reyes-Montes M. D. R. Estrada-Bárcenas D. A. Zancopé-Oliveira R. M. Rodríguez-Arellanes G. Ramírez J. A. (2022). Considerations about the geographic distribution of Histoplasma species. *Appl. Environ. Microbiol.* 88:e0201021. 10.1128/aem.02010-21 35262368 PMC9004366

[B185] The Integrative Hmp (iHMP) Research Network Consortium (2019). The integrative human microbiome project. *Nature* 569 641–648. 10.1038/s41586-019-1238-8 31142853 PMC6784865

[B186] Tomczyk-Żak K. Zielenkiewicz U. (2016). Microbial diversity in caves. *Geomicrobiol. J.* 33 20–38. 10.1080/01490451.2014.1003341

[B187] Turrini P. Tescari M. Visaggio D. Pirolo M. Lugli G. A. Ventura M. (2020). The microbial community of a biofilm lining the wall of a pristine cave in Western New Guinea. *Microbiol. Res.* 241:126584. 10.1016/j.micres.2020.126584 32882535

[B188] van Spanning R. J. Guan Q. Melkonian C. Gallant J. Polerecky L. Flot J. F. (2022). Methanotrophy by a *Mycobacterium* species that dominates a cave microbial ecosystem. *Nat. Microbiol.* 7 2089–2100. 10.1038/s41564-022-01252-3 36329197

[B189] Vanderwolf K. J. Malloch D. McAlpine D. F. Forbes G. J. (2013). A world review of fungi, yeasts, and slime molds in caves. *Int. J. Speleol.* 42 77–96. 10.5038/1827-806X.42.1.9

[B190] Vardeh D. P. Woodhouse J. N. Neilan B. A. (2018). Microbial diversity of speleothems in two Southeast Australian limestone cave arches. *J. Cave Karst Stud.* 80 121–132. 10.4311/2017MB0119

[B191] Vos M. Wolf A. B. Jennings S. J. Kowalchuk G. A. (2013). Micro-scale determinants of bacterial diversity in soil. *FEMS Microbiol. Rev.* 37 936–954. 10.1111/1574-6976.12023 23550883

[B192] Waite D. W. Vanwonterghem I. Rinke C. Parks D. H. Zhang Y. Takai K. (2017). Comparative genomic analysis of the class Epsilonproteobacteria and proposed reclassification to Epsilonproteobacteria (phyl. nov.). *Front. Microbiol.* 8:682. 10.3389/fmicb.2017.00682 28484436 PMC5401914

[B193] Wang D. Wang Y. Liu L. Chen Y. Wang C. Xu X. (2022). Niche differentiation and symbiotic association among ammonia/nitrite oxidizers in a full-scale rotating biological contactor. *Water Res.* 225:119137. 10.1016/j.watres.2022.119137 36198208

[B194] Wang X. W. Sun Z. Jia H. Michel-Mata S. Angulo M. T. Dai L. (2024). Identifying keystone species in microbial communities using deep learning. *Nat Ecol Evol.* 8 22–31. 10.1038/s41559-023-02250-2 37974003 PMC12125608

[B195] Wang Y. Zhao Y. Bollas A. Wang Y. Au K. F. (2021). Nanopore sequencing technology, bioinformatics and applications. *Nat. Biotechnol.* 39 1348–1365. 10.1038/s41587-021-01108-x 34750572 PMC8988251

[B196] Waring C. L. Hankin S. I. Griffith D. W. Kertesz M. A. Kobylski V. Wilson N. L. (2017). Seasonal total methane depletion in limestone caves. *Sci. Rep.* 7:8314. 10.1038/s41598-017-07769-6 28814720 PMC5559484

[B197] Watson T. H. Baldini J. U. L. (2024). Martian cave detection via machine learning coupled with visible light imagery. *Icarus* 411:115952. 10.1016/j.icarus.2024.115952

[B198] Weinroth M. D. Belk A. D. Dean C. Noyes N. Dittoe D. K. Rothrock M. J. (2022). Considerations and best practices in animal science 16S ribosomal RNA gene sequencing microbiome studies. *J. Anim. Sci.* 100:skab346. 10.1093/jas/skab346 35106579 PMC8807179

[B199] White W. B. Culver D. C. (2019). “Cave, definition of,” in *Encyclopedia of caves*, 3rd Edn, eds White W. B. Culver D. C. Pipan T. (Cambridge, MA: Academic Press), 255–259.

[B200] Wiseschart A. Mhuantong W. Tangphatsornruang S. Chantasingh D. Pootanakit K. (2019). Shotgun metagenomic sequencing from Manao-Pee cave, Thailand, reveals insight into the microbial community structure and its metabolic potential. *BMC Microbiol.* 19:144. 10.1186/s12866-019-1521-8 31248378 PMC6598295

[B201] Wood-Charlson E. M. Anubhav K. Auberry D. Blanco H. Borkum M. I. Corilo Y. E. (2020). The national microbiome data collaborative: Enabling microbiome science. *Nat. Rev. Microbiol.* 18 313–314. 10.1038/s41579-020-0377-0 32350400

[B202] Wu Y. Tan L. Liu W. Wang B. Wang J. Cai Y. (2015). Profiling bacterial diversity in a limestone cave of the western Loess Plateau of China. *Front. Microbiol.* 6:244. 10.3389/fmicb.2015.00244 25870592 PMC4378288

[B203] Zada S. Sajjad W. Rafiq M. Ali S. Hu Z. Wang H. (2022). Cave Microbes as a Potential Source of Drugs Development in the Modern Era. *Microb. Ecol.* 84 676–687. 10.1007/s00248-021-01889-3 34693460 PMC8542507

[B204] Zerkle A. L. Jones D. S. Farquhar J. Macalady J. L. (2016). Sulfur isotope values in the sulfidic Frasassi cave system, central Italy: A case study of a chemolithotrophic S-based ecosystem. *Geochim. Cosmochim. Acta* 173 373–386. 10.1016/j.gca.2015.10.028

[B205] Zhang Y. Thompson K. N. Branck T. Yan Y. Nguyen L. H. Franzosa E. A. (2021). Metatranscriptomics for the human microbiome and microbial community functional profiling. *Annu. Rev. Biomed. Data Sci.* 4 279–311. 10.1146/annurev-biodatasci-031121-103035 34465175

[B206] Zhao R. Wang H. Yang H. Yun Y. Barton H. A. (2017). Ammonia-oxidizing *Archaea* dominate ammonia-oxidizing communities within alkaline cave sediments. *Geomicrobiol. J.* 34 511–523. 10.1080/01490451.2016.1225861

[B207] Zhu H. Z. Jiang C. Y. Liu S. J. (2022). Microbial roles in cave biogeochemical cycling. *Front. Microbiol.* 13:950005. 10.3389/fmicb.2022.950005 36246268 PMC9554484

[B208] Zhu H. Z. Zhang Z. F. Zhou N. Jiang C. Y. Wang B. J. Cai L. (2021). Bacteria and metabolic potential in karst caves revealed by intensive bacterial cultivation and genome assembly. *Appl. Environ. Microbiol.* 87 e02440-20. 10.1128/AEM.02440-20 33452024 PMC8105019

