# Supplementary material for: The geomicrobiology of limestone, sulfuric acid speleogenetic, and volcanic caves: basic concepts and future perspectives
Source: Front Microbiol. 2024 Mar 20;15:1370520. doi: 10.3389/fmicb.2024.1370520 (PMC10987966; doi:10.3389/fmicb.2024.1370520)
Supplement: Supplementary file 2 [file Image_1.PDF]

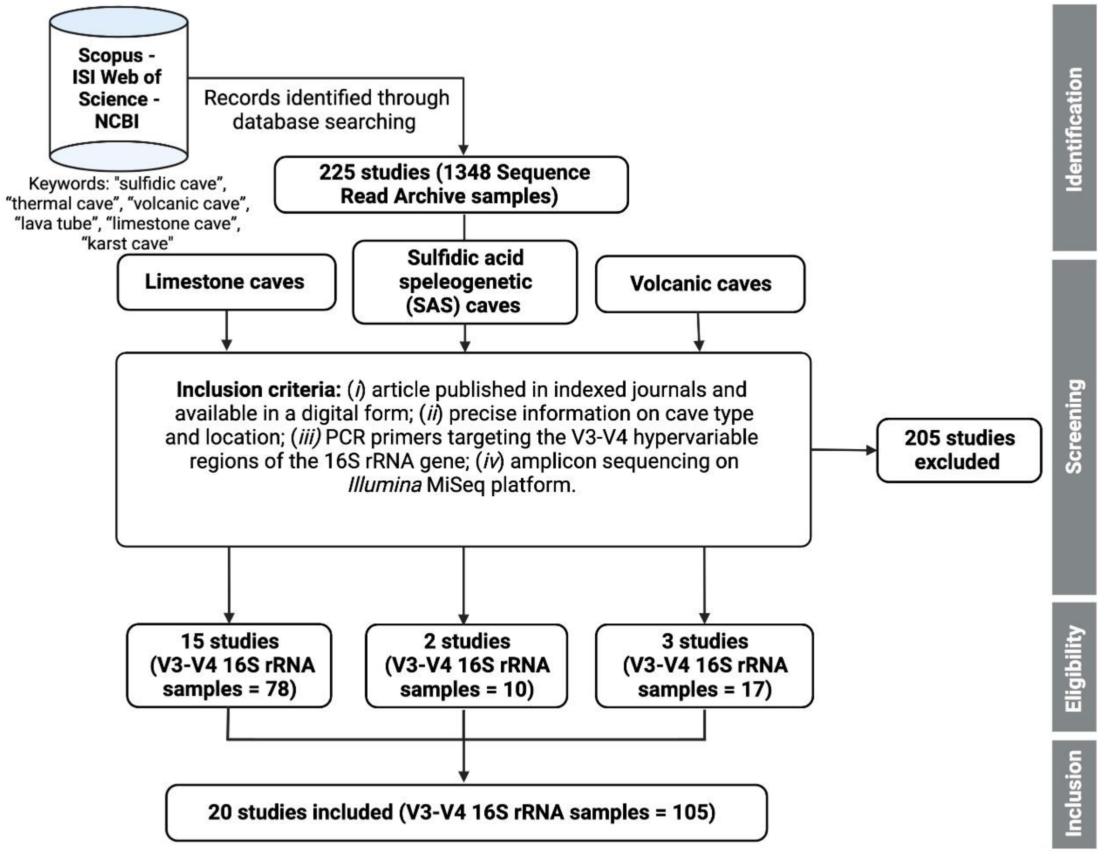

Fig. S1. Workflow illustrating the literature searches and the selection strategy of 16S rRNA amplicon-based cave microbiome studies. A search of cave microbiology studies in NCBI was started on December 2022 and finished on December 2023 upon database interrogation with the following keywords: “sulfidic cave”, “thermal cave”, “volcanic cave”, “lava tube”, “limestone cave”, “karst cave”. The search was performed to retrieve relevant microbiome studies related to the three main cave types. Literature was scrutinized and candidate articles were selected according to the following inclusion criteria: (*i*) precise information concerning the type of cave; (*ii*) amplicons had to be sequenced on the Illumina (MiSeq) sequencing platform; (*iii)* the PCR primers had to target the V3-V4 hypervariable regions of the 16S rRNA gene; (*iv*) article published on an online available indexed journal. Criteria of exclusion were: (*i*) other sequencing technologies; (*ii*) other hypervariable regions; (*iii*) an undefined type of cave. Summary data from the 19 selected studies, including 96 cave samples, are provided in Dataset S1.
